# Co_3_O_4_‑Promoted Cerium Oxide Catalyst for Efficient Catalytic *N*‑Alkylation of Amines with Alcohols

**DOI:** 10.1021/acsorginorgau.5c00069

**Published:** 2025-10-08

**Authors:** Jianyao Kou, Guangyao Yang, Zhixin Yu, Jiachao Liu, Yuhang Xu, Zhuo Xin, Yuxing Huang

**Affiliations:** † School of Pharmacy and Institute for Advanced Study, 47861 Nanchang University, Nanchang 330031, Jiangxi, P. R. China; ‡ School of Physics and Material Science, Nanchang University, Nanchang 330031, Jiangxi, P. R. China

**Keywords:** hydrogen borrowing, *N*-alkylation, synergistically catalyze, Ce^3+^/Ce^4+^ redox pairs, oxygen vacancies

## Abstract

The use of heterogeneous catalysts to generate amine compounds through anaerobic *N*-alkylation of alcohols via hydrogen transfer strategy is a highly promising synthetic strategy, as it can construct C–N bonds under relatively mild and green conditions. Moreover, amine compounds are widely used in the synthesis of pharmaceutical intermediates, agriculture and fine chemicals. In this work, we successfully constructed a novel Co_3_O_4_/CeO_2_ catalyst using the hydrothermal synthesis method and applied it to the *N*-alkylation reaction of alcohols, achieving up to 99% of the target product yield with broad substrate scope and high catalyst stability. The Ce^3+^/Ce^4+^ redox pairs and oxygen vacancies in the CeO_2_ support with highly dispersed Co species synergistically catalyze the hydrogen borrowing process of alcohols and amines to generate secondary amines with high activity and selectivity.

## Graphical abstract

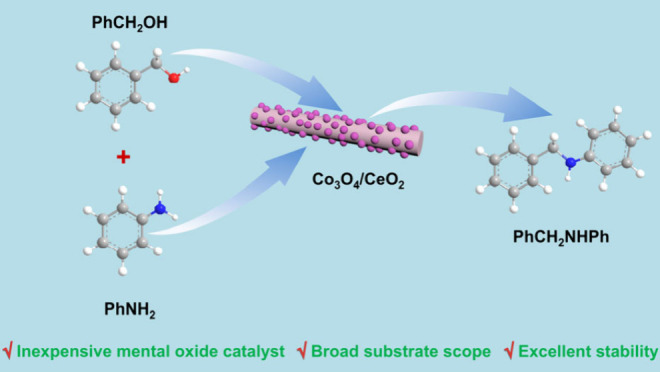

## Introduction

The borrowing hydrogen (BH) principle, also called hydrogen autotransfer (HAT) methodology, is a green and valuable strategy that combines transfer hydrogenation with intermediate reactions to synthesize various fine organic chemicals in one pot. The key to HAT is that the hydrogen from a donor molecule will be stored by the catalyst to be released in a final hydrogenation step, so this method avoids adding additional hydrogen sources and can coproduce dehydrogenation products, exhibiting excellent atomic utilization and synthesis efficiency.^1,2^ Generally, the *N*-alkylation of alcohols through the hydrogen borrowing (HB) reaction strategy involves three steps: (1) dehydrogenation of alcohols to form aldehydes, (2) aldehydes and amines condense to form imines, and (3) hydrogenation of imine to generate secondary amine (Scheme 1).^3–5^ And the byproduct of this method is only water, without the need for an external hydrogen source. It is widely regarded as a green and efficient universal strategy for synthesizing amine compounds.

**Scheme 1:**
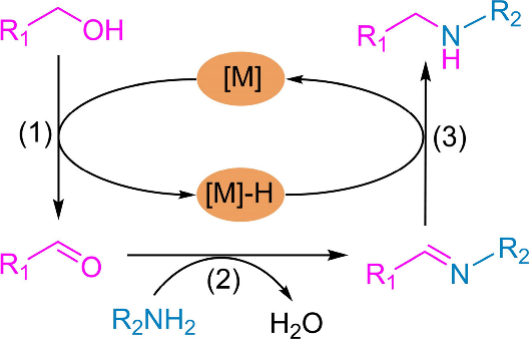
*N*-Alkylation of Alcohols through the Borrowing Hydrogen (BH) Principle

Amines are an important component in the syntheses of dyes, pharmaceuticals, agrochemicals, surfactants, rubber ingredients, and functional materials.^6,7^ Moreover, *N*-containing organic compounds are widely used as fine chemicals to construct various drug molecules and bioactive molecules, which maintain human life and health.^8–10^ The traditional methods for constructing C–N bonds in organic synthesis include the Buchwald-Hartwig reaction,^11^ hydroamination,^12^ and reductive amination.^13^ However, these processes often suffer from drawbacks, such as generation of inorganic salts and waste, excessive reducing agent and the use of unstable and expensive carbonyl compounds.^14,15^ The *N*-alkylation of alcohols and amines is a highly active and selective method for constructing a C–N bond through the oxidation process. This process relies on the HB strategy and has many advantages, such as the byproduct being only water, mild reaction conditions, and cheap and readily available raw materials, making it an efficient and green synthesis method for secondary amines.^16–18^

In homogeneous catalysis, many noble metal complexes such as Pd,^19^ Ru,^20^ Ir,^21^ etc. have been used to achieve high activity and selectivity in the *N*-alkylation reaction of alcohols. However, noble metals are expensive and difficult to widely apply on a large scale. In recent studies, first transition metals such as Fe,^22^ Ni,^23^ and Co^24^ have also been used to catalyze the HB reaction of alcohols and amines, but typically require high catalyst loadings and harsh reaction conditions. More importantly, homogeneous catalysts are difficult to recycle and reuse, greatly causing environmental pollution and resource waste. On the contrary, heterogeneous catalysts have the advantages of low cost, easy recovery, and good stability. In the past decade, especially transition-metal based catalysts have been used in HB reactions, mainly including supported Cu,^25,26^ Co,^27^ Fe,^28^ Ni^29^ based metal catalysts, which exhibit good activity and stability. In addition, metal oxide catalysts are also used for *N*-alkylation of amines.^30^ These supported catalysts can enhance substrate adsorption and H activation by regulating the interaction between the metal nanoparticles and the support.^31^ On one hand, due to the complex and diverse active sites on the surface of supported heterogeneous catalysts, it is difficult to control the selectivity of the reaction. On the other hand, metal nanoparticles are prone to aggregation during the reaction process, which reduces the reaction activity.^32^

Therefore, from the perspective of green sustainable development and atom economy, it is crucial to develop an efficient, inexpensive, and readily available heterogeneous catalyst for the *N*-alkylation of alcohols and amines under mild conditions. The *N*-alkylation reaction of alcohols following the HB reaction strategy is a cascade reaction step as shown in Scheme 1, which needs both oxidation and reduction functions of the catalyst, which is challenging for a heterogeneous catalyst. At present, CeO_2_-based catalysts have been successfully applied in the oxidation coupling of alcohols and amines to produce imine.^33–36^ This suggests that CeO_2_ is a promising candidate for further modification to achieve *N*-alkylation of alcohols via the HB strategy. The loading of transition metals on the CeO_2_ support is expected to achieve further hydrogenation of imines to amines. To the best of our knowledge, there are only two reports of CeO_2_ based composite catalysts in the HB reactions of alcohols and amines. Shi et al. first reported the HB reaction of alcohol and amine catalyzed by Cu/CeO_2_ composite catalyst. The Lewis acid base pairs at the interface of the Cu/CeO_2_ catalyst are proposed to be the key for the success of the *N*-alkylation reaction.^37^ Later on, Chen et al. successfully applied Ru/CeO_2_ in the *N*-alkylation reaction of alcohols and amines.^38^ However, these reports suffer from selectivity issues. To solve the problem, an ideal catalyst should be excellent in hydrogen transfer to ensure the full conversion of the imine intermediate.

Based on this, we utilize the oxygen vacancies in CeO_2_ to anchor Co species, which has been proven to be a good hydrogen transfer agent. At the same time, the oxygen vacancy caused Ce^3+^/Ce^4+^ redox pairs of CeO_2_ to contribute to the adsorption and activation of substrates. To our delight, the nanorod-shaped Co_3_O_4_/CeO_2_ catalyst we designed, which is rich in oxygen vacancies, with highly dispersed Co_3_O_4_ and Ce^3+^/Ce^4+^ redox pairs demonstrated excellent catalytic activity, selectivity, and stability in the *N*-alkylation reaction of alcohols and amines. Under relatively mild reaction conditions, the catalyst exhibits a target product yield of up to 99% in the *N*-alkylation of alcohols, and the yield remains above 94% after 6 reaction cycles, demonstrating excellent stability, thanks to the abundant Ce^3+^/Ce^4+^ active sites on the catalyst surface and the dispersion and anchoring effect of oxygen vacancies on Co_3_O_4_.

## Results and Discussion

X-ray diffraction (XRD) was used to investigate the crystal structure of the synthesized CeO_2_ without oxygen vacancies (denoted as N-CeO_2_), CeO_2_ containing oxygen vacancies (denoted as CeO_2_) and Co_3_O_4_/CeO_2_ (Figure 1a). The XRD patterns of N-CeO_2_ and CeO_2_ exhibit the same diffraction peaks at 28.5°, 33.1°, 47.5°, 56.3°, 59.1°, 69.4°, 76.7°, 79.1° and 88.4°, corresponding to (111), (200), (220), (311), (222), (400), (331), (420) and (422) of face-centered cubic CeO_2_ (a = b = c = 5.41 Å, α = β = γ = 90°, PDF 43–1002).^39^ It is worth noting that after loading with cerium oxide as a carrier there is almost no diffraction peak related to Co species in the XRD results of Co_3_O_4_/CeO_2_, indicating that the relevant Co species are highly dispersed on the surface of CeO_2_. To further determine the morphology of the Co_3_O_4_/CeO_2_ catalyst and the form of Co species, transmission electron microscopy (TEM) characterization was conducted. Figure 1b reveals that the Co_3_O_4_/CeO_2_ catalyst has a typical rod-shaped structure with a length of about 70 nm. The existence of a very obvious crystal plane boundary between Co_3_O_4_ and CeO_2_ can be clearly observed in Figure 1c. Moreover, Figure 1c also shows fringe distances of 0.466 nm, corresponding to the (111) crystal plane of Co_3_O_4_,^40,41^ while the fringe distances of 0.270 nm represent the (200) crystal plane of CeO_2_.^42,43^ Energy-dispersive spectroscopy (EDS) mapping of Co_3_O_4_/CeO_2_ showed a uniform distribution of Co, O and Ce elements (Figure 1d-g), which is consistent with the results of XRD (Figure 1a). The highly dispersed Co_3_O_4_ loaded on CeO_2_ plays a significant role in the high activity in the *N*-alkylation of alcohols.

**Figure 1:**
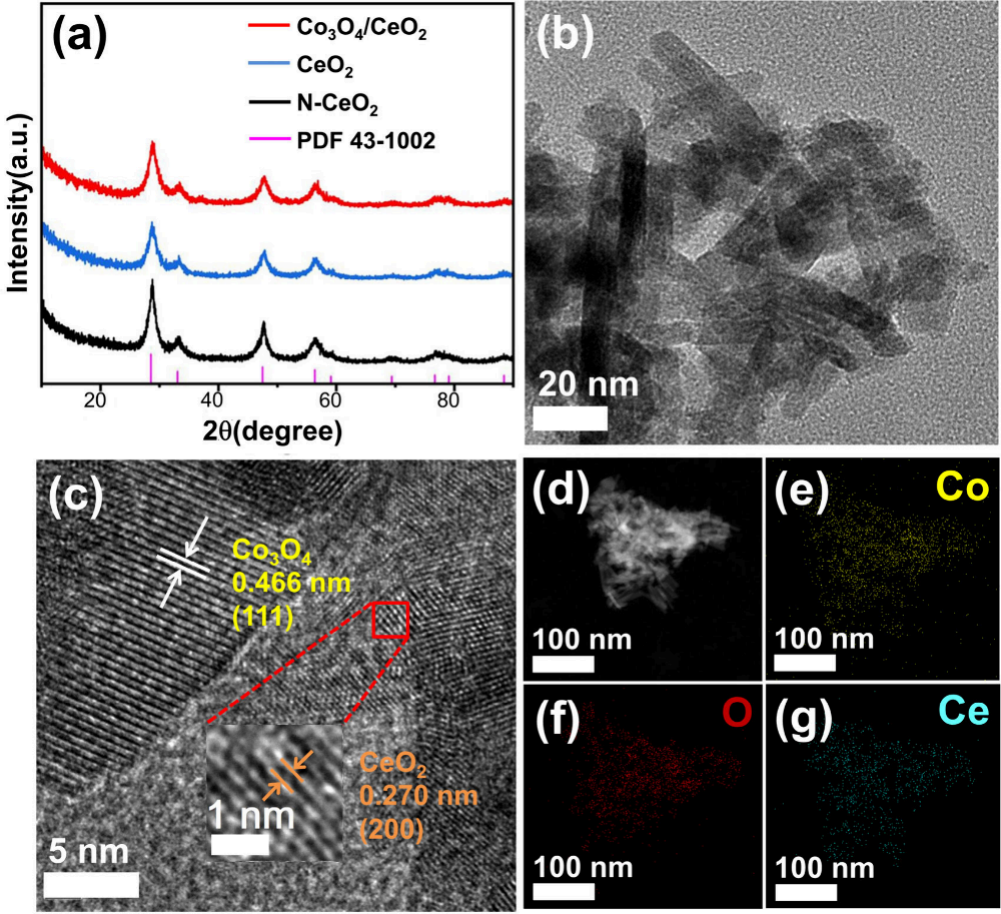
(a) XRD spectra of N-CeO_2_, CeO_2_ and Co_3_O_4_/CeO_2_, (b, c) TEM of Co_3_O_4_/CeO_2_ and (d–g) EDS mapping images of Co_3_O_4_/CeO_2_.

X-ray photoelectron spectroscopy (XPS) characterization was further applied to probe the elemental composition and surface chemical states of CeO_2_ and Co_3_O_4_/CeO_2_. The survey XPS full spectrum investigation in Figure 2a shows that CeO_2_ consists of Ce, O, and C elements, while the Co element was also found in Co_3_O_4_/CeO_2_ in addition to Ce, O, and C. This is consistent with the results of EDX mapping (Figure 1d-g). Based on the binding energy of C 1s (284.8 eV), the binding energies of other spectra have been corrected. Figure 2b shows Co 2p XPS spectra of Co_3_O_4_/CeO_2_, where the peaks at 776–784 eV and 792–799 eV were ascribed to Co 2p_3/2_ and Co 2p_1/2_, while the presence of two satellite peaks confirms the existence of Co^2+^ and Co^3+^. The binding energies of Co^2+^ on Co 2p_3/2_ and Co 2p_1/2_ are 781.1 and 796.2 eV, respectively. The binding energies of Co^3+^ on Co 2p_3/2_ and Co 2p_1/2_ are 779.6 and 794.6 eV, respectively.^44,45^ This confirms that Co_3_O_4_ is successfully loaded on CeO_2_. It is worth noting that the Co^2+^/Co^3+^ ratio of Co_3_O_4_/CeO_2_ catalyst was determined to be 0.60 by fitting the peak area, which is higher than that of Co^2+^/Co^3+^ in pristine Co_3_O_4_ (1.0).^46^ This is due to the reduction of the Co species by NaBH_4_ during catalyst preparation. Figure 2c shows that the O 1s XPS spectrum can be divided into three peaks through fitting, namely, lattice oxygen (∼529.5 eV, denoted as O_L_), oxygen vacancy (∼530.6 eV, denoted as O_V_), and chemisorbed oxygen (∼531.8 eV, denoted as O_C_).^47–50^ The molar ratios of O_V_:*O*_total_ in Co_3_O_4_/CeO_2_ catalyst was determined to be 11.5% by calculating the peak area through integration, which is lower than CeO_2_ catalyst (20.6%). This could be attributed to the occupation of oxygen vacancies by the highly dispersed Co species, which increased the number of metal active sites to promote the hydrogen transfer process. Figure 2d shows the Ce 3d XPS spectrum of the CeO_2_, which can be fitted into eight peaks, located at 882.4, 889.0, 898.3, 900.9, 907.3, and 916.7 eV are attributed to Ce 3d_5/2_ and 3d_3/2_ of the Ce^4+^. While 885.3 and 903.5 eV represent the Ce 3d_5/2_ and 3d_3/2_ of the Ce^3+^.^51,52^ The corresponding Ce^3+^ and Ce^4+^ species also exist in CeO_2_. The ratio of Ce^3+^/Ce^4+^ decreases after loading Co_3_O_4_, and the content of Ce^3+^ represents to some extent the number of oxygen vacancies, which also proves that highly dispersed Co species anchor on the oxygen vacancy and further enhance the stability of the catalyst. To further demonstrate the existence of oxygen vacancies, we conducted electron paramagnetic resonance (EPR) testing (Figure S1). It can be observed that no signal appears in N-CeO_2_. However, CeO_2_ has a clear paramagnetic symmetry signal at g = 2.003, indicating the presence of oxygen vacancies. And after loading Co_3_O_4_, the signal at g = 2.003 weakened, indicating a decrease in oxygen vacancy concentration. This is due to the high dispersion of Co species on the CeO_2_ surface, which is consistent with the results of XPS (Figure 2c, d).

**Figure 2:**
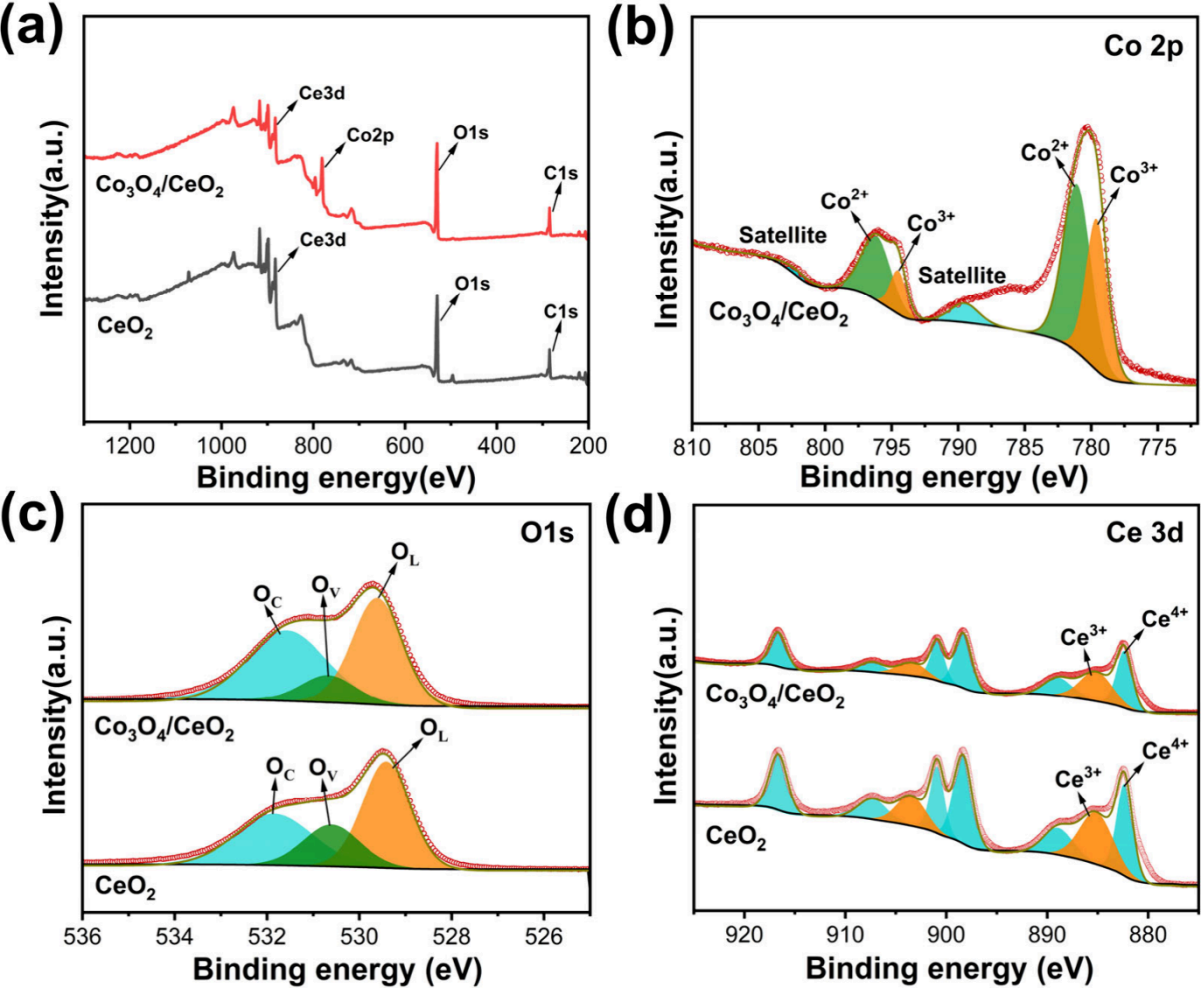
XPS characterization of CeO_2_ and Co_3_O_4_/CeO_2_: (a) Survey spectrum; (b) Co 2p; (c) O 1s; (d) Ce 3d.

The study of Co_3_O_4_/CeO_2_ catalytic activity was initiated using the reaction between benzyl alcohol and aniline as the model reaction to optimize. First, the influence of Co loading on *N*-alkylation reaction was investigated (Figure S2). Among them, when the Co loading is 20 wt %, the highest yield is 63%. Afterward, the influence of other reaction conditions on the experimental results was explored (Table 1). We tried N-CeO_2_ as the catalyst, but the yield was merely 5% (Entry 1). When CeO_2_ is used as a catalyst, the product yield significantly increases to 59% (Entry 2), and further increases to 99% (Entry 3) after loading Co_3_O_4_, and the appropriate amount of catalyst is 5 mg (Entry 3–5), indicating that both oxygen vacancies and Co_3_O_4_ were essential for the reaction. Next, when a base such as KOH was introduced into the reaction system, the reaction achieved an excellent yield (entry 4 vs entry 9). But the use of other bases such as NaOH, K_2_CO_3_, or Na_2_CO_3_ resulted in no reaction (Entry 6–8). In addition, in the presence of only the Co­(NO_3_)·6H_2_O precursor, **3a** was generated (Entry 10). Under the catalysis of KOH, a 34% yield of **3a** can be obtained (Entry 11). Then in the presence of both Co­(NO_3_)·6H_2_O and KOH, only 8% **3a** can be obtained (Entry 12). The above results indicate that KOH plays an important role in the *N*-alkylation of benzyl alcohol and aniline. This result is also similar to related reports.^53–55^ The solvent also affects the performance of the *N*-alkylation reaction. Changing the solvent from toluene to DMF or DMSO resulted in no reaction (Entry 13–14). When the reaction temperature drops to 150 °C, no target product is generated (Entry 15). Finally, when the benzyl alcohol: aniline ratio was reduced from 4:1 to 2:1, the corresponding yield decreased from 99% to 63% (entry 4 vs 16).

**Table 1:**
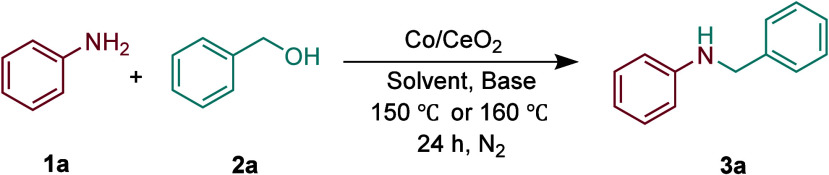
Screening of Reaction Conditions for the *N*-Alkylation of Aniline (1a) with Benzyl Alcohol (2a)^a^

Entry	Catalyst	Dosing (mg)	Base	Solvent	Yield^b^ (%)
1	N-CeO_2_	5	KOH	Toluene	13%
2	CeO_2_	5	KOH	Toluene	59%
3	Co_3_O_4_/CeO_2_	10	KOH	Toluene	95%
**4**	**Co**_**3**_ **O**_**4**_ **/CeO**_**2**_	**5**	**KOH**	**Toluene**	**99%**
5	Co_3_O_4_/CeO_2_	3	KOH	Toluene	26%
6	Co_3_O_4_/CeO_2_	5	Na_2_CO_3_	Toluene	N.R.
7	Co_3_O_4_/CeO_2_	5	K_2_CO_3_	Toluene	N.R.
8	Co_3_O_4_/CeO_2_	5	NaOH	Toluene	N.R.
9	Co_3_O_4_/CeO_2_	5	—	Toluene	N.R.
10	—		KOH	Toluene	34%
11	Co(NO_3_)_2_·6H_2_O	5	—	Toluene	N.R.
12	Co(NO_3_)_2_·6H_2_O	5	KOH	Toluene	8%
13	Co_3_O_4_/CeO_2_	5	KOH	DMF	N.R.
14	Co_3_O_4_/CeO_2_	5	KOH	DMSO	N.R.
15^c^	Co_3_O_4_/CeO_2_	5	KOH	Toluene	N.R.
16^d^	Co_3_O_4_/CeO_2_	5	KOH	Toluene	63%

a Reaction conditions were as follows: **1a** (0.125 mmol, 1.0 equiv), **2a** (0.5 mmol, 4.0 equiv), base (0.125 mmol, 1.0 equiv) and catalyst (x mg) were reacted in 1 mL of solvent at 160 °C under N_2_ for 24 h.

b GC-MS yields, using *n*-dodecane as the internal standard.

c Reaction temperature is 150 °C.

d **2a** (0.25 mmol, 2.0 equiv).

Under the optimized conditions, the substrate scope of benzyl alcohol and its derivatives in the *N*-alkylation was investigated (Scheme 2). The *N*-alkylation reactions of aniline with various alcohols were explored. Benzyl alcohols with both electron-withdrawing and electron-donating groups were transformed into the desired products with good-to-excellent yields, such as alkyl, -OMe, -F, -Cl, and -Br (**3a-3e, 3h-3j, 3n**), further demonstrating a nonsignificant electronic effect of benzyl alcohol derivatives. Then, we attempted *N*-alkylation reactions of pyridine methanol, thiophene methanol, and furan methanol (**3k**–**3m**). Among them, both pyridine methanol and thiophene methanol were coupled with aniline to provide the corresponding heterocyclic secondary amines in up to an 84% yield. However, furan methanol achieved only a 30% yield. We speculate that this is related to the adsorption configuration of oxygen in the furan ring on the catalyst surface.

**Scheme 2:**
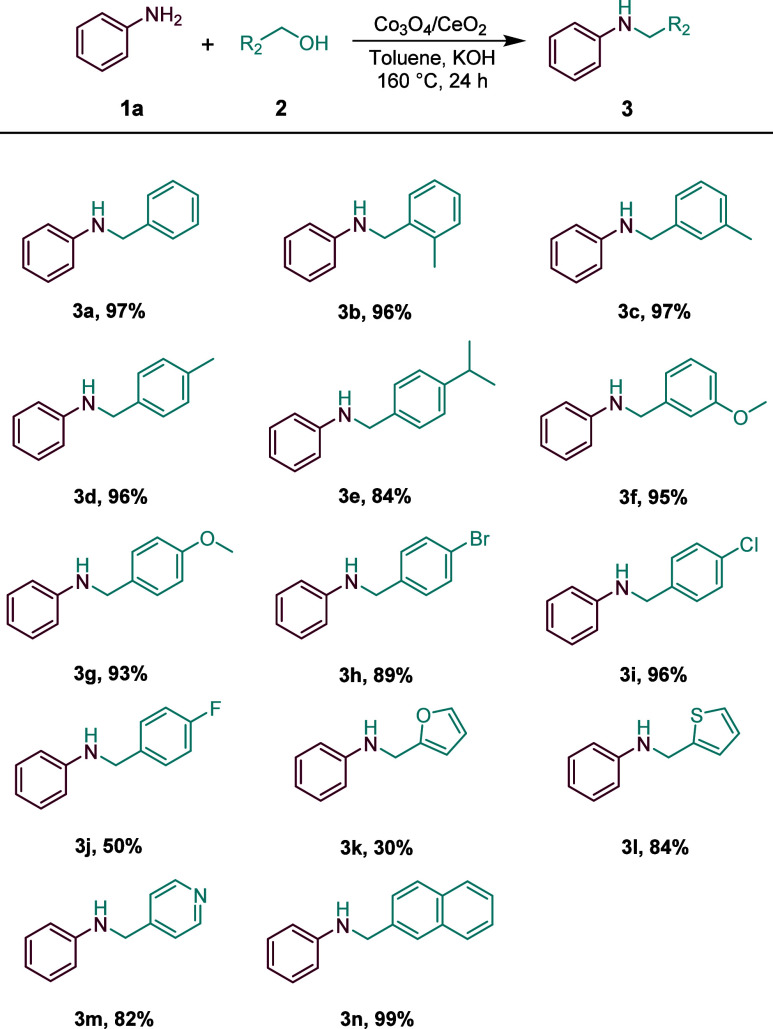
Co_3_O_4_/CeO_2_–Catalyzed *N*-Alkylation of Aniline with Alcohols^a^

a Conditions: **1a** (0.125 mmol), **2** (0.5 mmol, 4.0 equiv), Co_3_O_4_/CeO_2_ (5 mg), KOH (0.125 mmol, 1.0 equiv) and toluene (1 mL) were reacted in N_2_ at 160 °C for 24 h. Isolated yields.

Next, we explored the scope of amines, and a series of substituted and functionalized *N*-benzylanilines was prepared. As presented in Scheme 3, the benzyl alcohol reacting with the substrates with electron-donating groups such as methyl-, methoxy-, and *tert*-butyl- or substrates with electron-withdrawing groups such as F-, Cl- and Br- also provided the corresponding products in excellent yield (**4a**–**4i**). Especially, *ortho*-methyl and *ortho*-methoxy substituents all achieved good yields (**4c, 4f**). Additionally, 1,2-diaminobenzene can get a high yield of 84% (**4j**), However, the benzylic and aliphatic primary amines afforded only the imine products (**4k, 4l**). This indicates that the corresponding imine cannot be hydrogenated during the reaction.

**Scheme 3:**
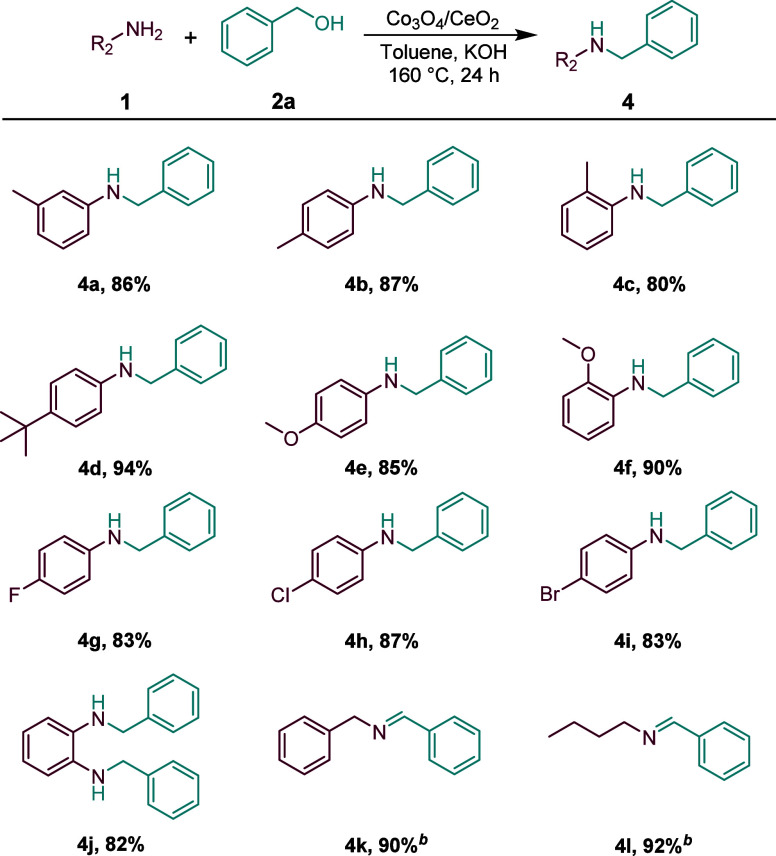
Co_3_O_4_/CeO_2_–Catalyzed *N*-Alkylation of Amines with Benzyl Alcohol^a^

a Conditions: **1** (0.125 mmol), **2a** (0.5 mmol, 4.0 equiv), Co_3_O_4_/CeO_2_ (5 mg), KOH (0.125 mmol, 1.0 equiv) and toluene (1 mL) were reacted in N_2_ at 160 °C for 24 h. Isolated yields.
b GC-MS yields, using *n*-dodecane as the internal standard.

To demonstrate the practical utility of the method for synthesizing *N*-benzyl aniline, we conducted a scaling up experiment. Surprisingly, the reaction could be easily scaled up, resulting in the desired product **3a** in 81% yield (Figure 3a). The recyclability of the Co_3_O_4_/CeO_2_ catalyst in the *N*-alkylation reaction of benzyl alcohol with aniline was investigated. Figure 3b demonstrates that 94% product yield can still be achieved after the sixth run. Moreover, the XRD spectra of the catalyst before and after cycling showed that there was no significant change (Figure 3c), which indicates the robustness of the Co_3_O_4_/CeO_2_ catalyst and its potential for possible industrial application.

**Figure 3:**
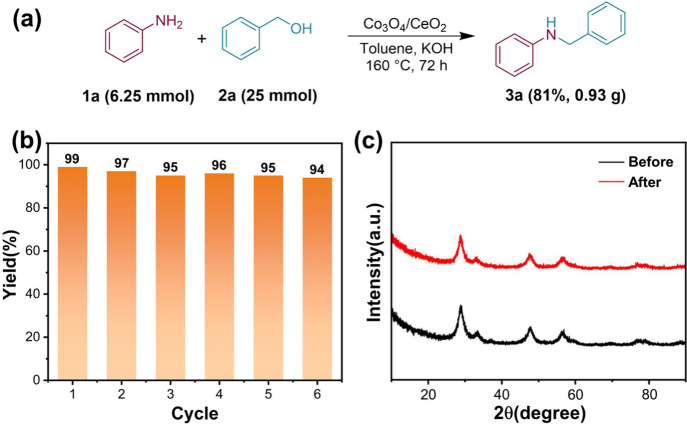
(a) Scale-up experiment. Conditions: **1a** (6.25 mmol), **2a** (25 mmol, 4.0 equiv), Co_3_O_4_/CeO_2_ (250 mg), KOH (6.25 mmol, 1.0 equiv) and toluene (50 mL) were reacted in N_2_ at 160 °C for 72 h. Isolated yields. (b) Reusability of Co_3_O_4_/CeO_2_ until the sixth cycle in *N*-alkylation reaction of benzyl alcohol with aniline. (c) XRD spectra of Co_3_O_4_/CeO_2_ before and after cycling.

In order to further investigate the reaction mechanism of *N*-alkylation of benzyl alcohol and aniline, we conducted a radical quenching experiment (Scheme S2). The results showed that using 2,2,6,6-tetramethyl-1piperidinyloxy (TEMPO, 1.0 equiv) as a radical scavenger, the yield of *N*-benzyl aniline (**3a**) could still reach 90% with Co_3_O_4_/CeO_2_ as the catalyst. This indicates that the reaction does not involve radical reaction process. According to the previous report,^34,35,56–59^ the possible reaction mechanism of *N*-alkylation reaction of benzyl alcohol and aniline catalyzed by Co_3_O_4_/CeO_2_ was proposed (Figure 4). First, in the presence of KOH, benzyl alcohol undergoes deprotonation to generate a benzyl alkoxide intermediate. Then the generated oxygen vacancies in Co_3_O_4_/CeO_2_ catalyst served as the Lewis acid site could adsorb the benzyl alkoxide and converted it into benzaldehyde. Meanwhile, active H species could produce and transfer to Co species to form Co–H. Then, with the assistance of KOH, imine can be easily formed from benzaldehyde and aniline. Finally, the Co–H species reduced the C = N bond of imine to generate the desired product.

**Figure 4:**
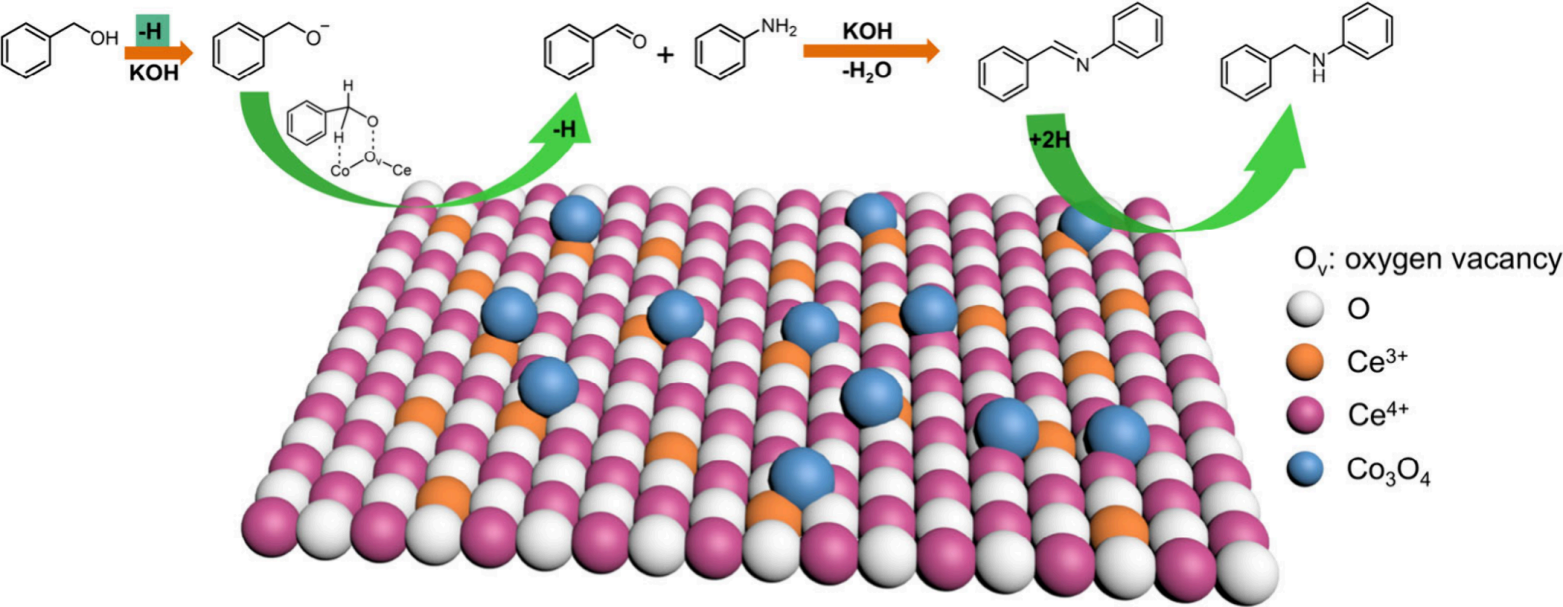
Proposed mechanism for the Co_3_O_4_/CeO_2_-catalyzed *N*-alkylation reaction.

## Conclusion

In summary, a novel nanorod Co_3_O_4_/CeO_2_ catalyst was designed and prepared. The anchoring effect of Co species by oxygen vacancies in CeO_2_ secured the high dispersion of Co_3_O_4_ on CeO_2_, which is a good hydrogen transfer agent. At the same time, the oxygen vacancy caused by the Ce^3+^/Ce^4+^ redox pairs of CeO_2_ contributes to the adsorption and activation of substrates. Based on this, a series of amine compounds was successfully synthesized with high efficiency and selectivity under relatively mild conditions using a hydrogen borrowing strategy by this catalyst. Recycling experiment results demonstrated the robustness of this catalyst. Therefore, this work provides an inexpensive, green, and efficient method for producing amine compounds, and offers a new insight for the rational design of metal loaded CeO_2_-based catalyst.

## Experimental Section

All solvents and chemicals were purchased from Greagent, Aladdin, Incochem or Adamas and used as received without any purification. ^1^H and ^13^C NMR spectra were recorded on a Varian 400 spectrometer using deuterated CDCl_3_ as a solvent. Chemical shifts are reported in ppm (δ) relative to internal tetramethylsilane (TMS, δ 0.0 ppm) or with the solvent reference relative to TMS employed as an internal standard (CDCl_3_, δ 7.26 ppm). The following abbreviations were used to identify the multiplicities: s = singlet, d = doublet, t = triplet, q = quartet, m = multiplet, b = broad, and all combinations thereof can be explained by their integral parts.

### Preparation of N-CeO_2_

Typically, 1.7 g of Ce­(NO_3_)_3_·6H_2_O was dissolved in 5 mL of deionized water to form solution A; meanwhile, 19.2 g of NaOH was dissolved in 75 mL of deionized water to form solution B. Then solutions A and B were mixed slowly and the mixture aged for 30 min under continuous stirring. Subsequently, the mixture was transferred to a 100 mL flask and reacted at 100 °C in an oil bath for 24 h under reflux condition. After cooling to room temperature, collect the product by centrifugation, the precipitate was collected and wash it with deionized water for several times, then dry it under vacuum at 60 °C overnight. Finally, the precursor was hydrothermal at 160 °C for 12 h and then washed three times with deionized water and vacuum-dried overnight at 60 °C to obtain vacancy free CeO_2_ (named N-CeO_2_).

### Preparation of CeO_2_ Nanorods

First, 1.7 g of Ce­(NO_3_)_3_·6H_2_O was dissolved in 5 mL of deionized water to form solution A, and 19.2 g of NaOH was dissolved in 75 mL of deionized water to form solution B. The solution A was slowly added to solution B under continuous stirring for 30 min. Subsequently, the mixture was aged at room temperature for 1 h. Next, the mixture was transferred to a 100 mL flask and reacted at 100 °C in an oil bath for 24 h under reflux condition. After the mixture cooled to room temperature, the precipitate was collected and washed alternately with deionized water and absolute ethanol three times, and then it was dried under vacuum at 60 °C overnight to obtain CeO_2_ nanorod.

### Preparation of Co_3_O_4_/CeO_2_ Nanorods

As shown in Scheme S1, first, 100 mg of the prepared CeO_2_ was dispersed in a 0.05 M solution of Co­(NO_3_)_2_·6H_2_O (98.8 mg in 6.8 mL of deionized water), followed by further ultrasonic treatment for 30 min. Then, a 0.02 M NaBH_4_ (51.6 mg in 68 mL deionized water) solution and a 0.01 M NaOH (27.3 mg in 68 mL deionized water) solution were mixed and added dropwise to the above solution at 80 °C in an oil bath while continuously stirring. After 2 h of reaction, the precipitate was collected and washed with deionized water several times, and then it was dried under vacuum at 60 °C overnight to obtain Co_3_O_4_/CeO_2_ solid powder.

### Catalytic Activity Test

The *N*-alkylation of alcohol was performed in a 10 mL screw cap reaction tube. Typically, KOH (0.125 mmol, 1.0 equiv) and Co_3_O_4_/CeO_2_ catalyst (5 mg) were charged into the reaction tube. The reaction tube was then introduced in a glovebox, where it was charged with alcohol (0.5 mmol, 4 equiv), aromatic amine (0.125 mmol, 1.0 equiv) and toluene (1 mL). The tube was sealed and taken out of the glovebox. The reaction tube was heated with an aluminum block to 160 °C and kept at this temperature for 24 h at a stirring speed of 900 rpm. At the end of the reaction, the reaction tube was cooled to room temperature, and the reaction mixture was sampled and subjected to GC-MS analysis. For the scope of substrates, the reaction mixture was purified using silica gel chromatography column and weighed to the isolated yield. ^1^H NMR and ^13^C NMR analyses were performed to confirm the structure. For the recycling test, the catalyst was recovered after the reaction and washed with ethyl acetate three times. The solid catalyst was directly placed in the reaction tube for the next run.

### Scale-Up Experiment of Synthesizing Compound 3a

The scale-up experiment of synthesizing **3a** was performed in a 100 mL screw cap reaction tube. Typically, KOH (6.25 mmol, 1.0 equiv) and Co_3_O_4_/CeO_2_ catalyst (250 mg) were charged into the reaction tube. The reaction tube was then introduced in a glovebox, where it was charged with benzyl alcohol (25 mmol, 4 equiv), aromatic amine (6.25 mmol, 1.0 equiv) and toluene (50 mL). The tube was sealed and taken out of the glovebox. The reaction tube was heated with an oil bath aluminum block to 160 °C and kept at this temperature for 72 h at a stirring speed of 900 rpm. At the end of the reaction, the reaction tube was cooled to room temperature. Then the catalyst was separated and washed with ethyl acetate three times. The reaction mixture was purified using silica gel chromatography column and weighed to the isolated yield. ^1^H NMR and ^13^C NMR analyses were performed to confirm the structure.

### Characterization for Catalysts

Powder X-ray diffraction (XRD) of the sample data was collected on a Shimadzu XRD-6100 X-ray diffractometer employing a CuKα radiation source and operated at 40 kV and 30 mA. The scanning rate was 0.05° s^–1^ from 10° to 80° for wide-angle XRD. Transmission electron microscopy (TEM) characterization was performed using a TECNAI F20 instrument operated at 200 kV from FEI Company. The point and line resolutions were 0.24 and 0.10 nm, respectively. X-ray photoelectron spectroscopy (XPS) was measured on a Thermo Fisher Scientific K-Alpha instrument. Analyze and process data using Avantage software. Electron paramagnetic resonance (EPR) characterization was performed using a E500–10/12 spectrometer.

## Supplementary Material



## Data Availability

The data underlying this study are available in the published article and its Supporting Information.

## Notes

Other experimental results, flowchart of catalyst preparation, NMR data and spectra of all compounds (PDF)
