# Supplementary material for: Co3O4‑Promoted Cerium Oxide Catalyst for Efficient Catalytic N‑Alkylation of Amines with Alcohols
Source: ACS Org Inorg Au. 2025 Oct 8;5(6):498–506. doi: 10.1021/acsorginorgau.5c00069 (PMC12679301; doi:10.1021/acsorginorgau.5c00069)
Supplement: Supplementary file 1 [file gg5c00069_si_001.pdf]

# Supporting Information

## $Co_3O_4$-promoted cerium oxide catalyst for efficient catalytic *N*-alkylation of amines with alcohols

Jianyao Kou[1], Guangyao Yang[2], Zhixin Yu[1], Jiachao Liu[2], Yuhang Xu[2], Zhuo Xin[1]*, Yuxing Huang[2]*

[1]School of Pharmacy and Institute for Advanced Study, Nanchang University, Nanchang 330031 Jiangxi, P. R. China.

[2]School of Physics and Material Science, Nanchang University, Nanchang 330031 Jiangxi, P. R. China.

Corresponding author E-mail: xinzhuo2@gmail.com; yuxinghuang2@gmail.com

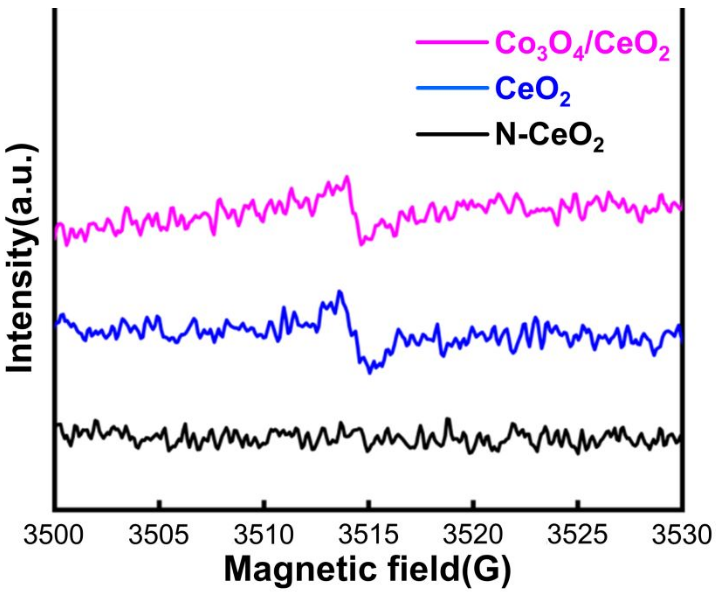


**Figure S1**. EPR spectra of N-$CeO_2$、$CeO_2$ and $Co_3O_4/CeO_2$

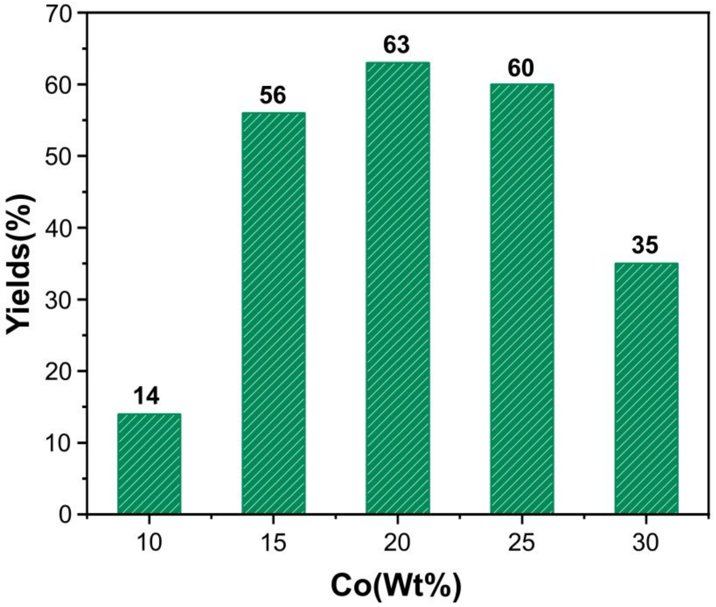


**Figure S2**. Reaction results of catalysts with different Co loadings. Reaction conditions were as follows: aniline (0.125 mmol, 1.0 equiv.), benzyl alcohol (0.5 mmol, 4.0 equiv.), KOH (0.125 mmol, 1.0 equiv.) and catalyst (5 mg) were reacted in 1 mL toluene at 160 °C under $N_2$ for 24 h. GC-MS yields, using *n*-dodecane as the internal standard.

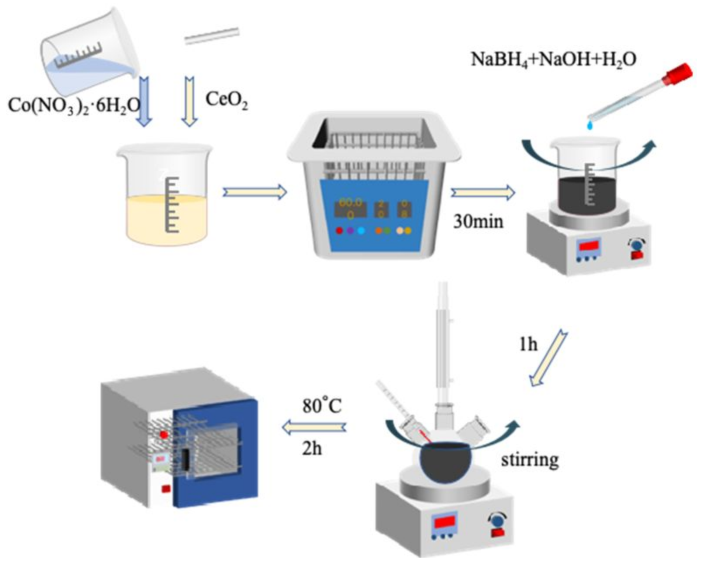


**Scheme S1**. Preparation process diagram of $Co_3O_4/CeO_2$

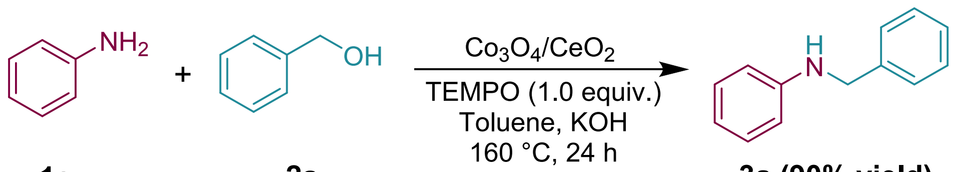


**1a** **2a** **3a (90% yield)**

**Scheme S2**. Radical quenching experiment. Conditions: **1a** (0.125 mmol, 1.equiv.), **2a** (0.5 mmol, 4.0 equiv.), $Co_3O_4/CeO_2$ (5 mg), KOH (0.125 mmol, 1.0 equiv.) and toluene (1 mL) were reacted in $N_2$ at 160 °C for 24 h. GC-MS yields, using *n*-dodecane as the internal standard.

## $^1$H and $^{13}$C NMR data of products

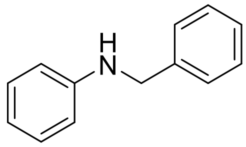


*N*-benzylaniline (3a)

The reaction was purified by silica gel column chromatography (petroleum ether/EtOAc 46:1).

The product was isolated (23.2 mg, 97%) as a colorless oil.

$^{1}H$ NMR (600 MHz, $CDCl_3$) δ 7.37 (dt, *J* = 15.2, 7.4 Hz, 4H), 7.29 (t, *J* = 7.2 Hz, 1H), 7.22 – 7.17 (m, 2H), 6.74 (t, *J* = 7.3 Hz, 1H), 6.66 (d, *J* = 7.6 Hz, 2H), 4.35 (s, 2H), 4.04 (s, 1H). $^{13}C\{^{1}H\}$ NMR (151 MHz, $CDCl_3$) δ 148.3, 139.6, 129.4, 128.7, 127.6, 127.3, 117.7, 113.0, 48.5. The spectral data are in agreement with previous report[1].

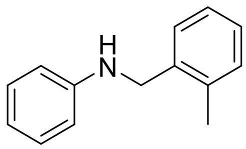

*N*-(2-methylbenzyl)aniline (3b)

The reaction was purified by silica gel column chromatography (petroleum ether/EtOAc 97:3). The product was isolated (23.7 mg, 96%) as an orange oil.

$^{1}H$ NMR (400 MHz, $CDCl_3$) δ 7.41 (d, *J* = 6.9 Hz, 1H), 7.33 – 7.20 (d, 5H), 6.81 (t, *J* = 7.3 Hz, 1H), 6.73 (d, *J* = 7.9 Hz, 2H), 4.34 (s, 2H), 2.43 (s, 3H). $^{13}C\{^{1}H\}$ NMR (101 MHz, $CDCl_3$) δ 148.2, 137.0, 136.4, 130.4, 129.3, 128.3, 127.4, 126.2, 117.6, 112.8, 46.4, 18.9. The spectral data are in agreement with previous report[1].

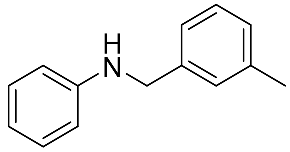

*N*-(3-methylbenzyl)aniline (3c)

The reaction was purified by silica gel column chromatography (petroleum ether/EtOAc 46:1). The product was isolated (23.7 mg, 96%) as an yellow oil.

$^1H$ NMR (400 MHz, $CDCl_3$) δ 7.24 – 7.08 (m, 6H), 6.74 (t, *J* = 7.4 Hz, 1H), 6.67 (d, *J* = 8.0 Hz, 2H), 4.30 (s, 2H), 2.36 (s, 3H). $^{13}C\{^1H\}$ NMR (101 MHz, $CDCl_3$) δ 148.1, 139.2, 138.3, 129.2, 128.5, 128.3, 128.0, 124.6, 117.6, 113.0, 48.4, 21.4. The spectral data are in agreement with previous report[1].

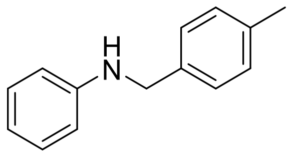

*N*-(4-methylbenzyl)aniline (3d)

The reaction was purified by silica gel column chromatography (petroleum ether/EtOAc 46:1). The product was isolated (23.7 mg, 96%) as an orange oil.

$^1H$ NMR (400 MHz, $CDCl_3$) δ 7.24 – 7.21 (m, 2H), 7.21 – 7.08 (m, 4H), 6.75 (t, *J* = 7.3 Hz, 1H), 6.68 (d, *J* = 7.9 Hz, 2H), 4.30 (s, 2H), 2.36 (s, 3H). $^{13}C\{^1H\}$ NMR (101 MHz, $CDCl_3$) δ148.1, 136.9, 136.3, 129.3, 129.2, 127.5, 117.6, 112.9, 48.2, 21.1. The spectral data are in agreement with previous report[1].

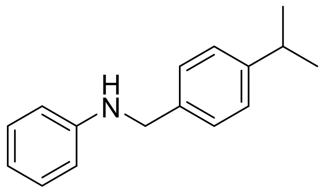

*N*-(4-isopropylbenzyl)aniline (3e)

The reaction was purified by silica gel column chromatography (petroleum ether/EtOAc 93:7). The product was isolated (23.7 mg, 84%) as a colorless oil.

$^{1}$H NMR (400 MHz, $CDCl_3$) δ 7.30 (d, *J* = 7.8 Hz, 2H), 7.19 (dd, *J* = 9.9, 7.9 Hz, 4H), 6.76 (d, *J* = 7.4 Hz, 1H), 6.70 (d, *J* = 7.9 Hz, 2H), 4.29 (s, 2H), 2.90 (d, *J* = 6.9 Hz, 1H), 1.25 (d, *J* = 7.0 Hz, 6H). $^{13}C\{^1H\}$ NMR (101 MHz, $CDCl_3$) δ 148.0, 148.0, 136.6, 129.2, 127.7, 126.7, 117.6, 112.9, 48.2, 33.8, 24.0. The spectral data are in agreement with previous report[2].

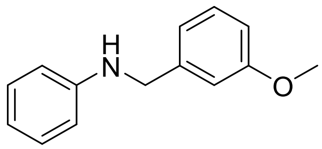

*N*-(3-methoxybenzyl)aniline (3f)

The reaction was purified by silica gel column chromatography (petroleum ether/EtOAc 46:1). The product was isolated (20.2 mg, 95%) as a colorless oil.

$^1H$ NMR (600 MHz, $CDCl_3$) δ 7.27 – 7.22 (m, 1H), 7.16 (t, *J* = 7.9 Hz, 2H), 7.00 – 6.89 (m, 2H), 6.81 (dd, *J* = 8.2, 2.2 Hz, 1H), 6.71 (t, *J* = 7.3 Hz, 1H), 6.63 (d, *J* = 7.8 Hz, 2H), 4.29 (s, 2H), 4.02 (s, 1H), 3.78 (s, 3H). $^{13}C\{^1H\}$ NMR (151 MHz, $CDCl_3$) δ 160.0, 148.2, 141.3, 129.7, 129.3, 119.8, 117.7, 113.1, 113.0, 112.7, 55.3, 48.4. The spectral data are in agreement with previous report[3].

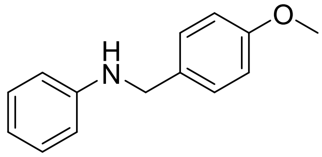

*N*-((4-methoxy)benzyl)aniline (3g)

The reaction was purified by silica gel column chromatography (petroleum ether/EtOAc 46:1). The product was isolated (24.8 mg, 93%) as a colorless oil.

$^{1}$H NMR (400 MHz, $CDCl_3$) δ 7.31 (d, *J* = 8.6 Hz, 2H), 7.23 – 7.14 (m, 2H), 6.89 (d, *J* = 8.7 Hz, 2H), 6.74 (t, *J* = 7.4 Hz, 1H), 6.66 (d, *J* = 7.5 Hz, 2H), 4.27 (s, 2H), 3.82 (s, 3H). $^{13}C\{^1H\}$ NMR (101 MHz, $CDCl_3$) δ 158.8, 148.0, 131.3, 129.2, 128.8, 117.6, 114.0, 113.0, 55.3, 47.9. The spectral data are in agreement with previous report[4].

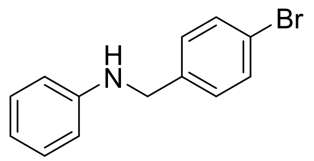


*N*-(4-bromobenzyl)aniline (3h)

The reaction was purified by silica gel column chromatography (petroleum ether/EtOAc 46:1). The product was isolated (29.0 mg, 89%) as a colorless oil.

$^1H$ NMR (600 MHz, $CDCl_3$) δ 7.46 (d, *J* = 8.2 Hz, 2H), 7.25 (s, 2H), 7.18 (t, *J* = 7.6 Hz, 2H), 6.74 (t, *J* = 7.3 Hz, 1H), 6.61 (d, *J* = 8.2 Hz, 2H), 4.30 (s, 2H), 4.07 (s, 1H). $^{13}C\{^1H\}$ NMR (151 MHz, $CDCl_3$) δ 147.9, 131.8, 129.4, 129.1, 118.0, 113.0, 47.8[1].

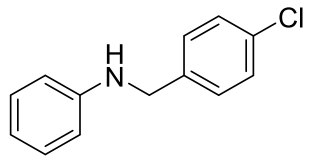


*N*-(4-chlorobenzyl)aniline (3i)

The reaction was purified by silica gel column chromatography (petroleum ether/EtOAc 46:1). The product was isolated (26.0 mg, 93%) as a colorless oil.

$^{1}H$ NMR (400 MHz, $CDCl_3$) δ 7.32 (s, 4H), 7.19 (t, *J* = 7.8 Hz, 2H), 6.75 (t, *J* = 7.4 Hz, 1H), 6.63 (d, *J* = 8.3 Hz, 2H), 4.32 (s, 2H). $^{13}C\{^{1}H\}$ NMR (101 MHz, $CDCl_3$) δ 147.7, 137.9, 132.9, 129.3, 128.7, 128.7, 117.9, 113.0, 47.7. The spectral data are in agreement with previous report[1].

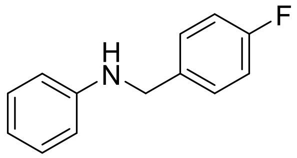


*N*-(4-fluorobenzyl)aniline (3j)

The reaction was purified by silica gel column chromatography (petroleum ether/EtOAc 46:1). The product was isolated (12.6 mg, 50%) as a yellow oil.

$^{1}H$ NMR (600 MHz, $CDCl_3$) δ 7.38 – 7.31 (m, 2H), 7.23 – 7.16 (m, 2H), 7.04 (t, *J* = 8.7 Hz, 2H), 6.77 – 6.72 (m, 1H), 6.64 (d, *J* = 7.7 Hz, 2H), 4.32 (s, 2H), 4.01 (s, 1H). $^{13}C\{^{1}H\}$ NMR (151 MHz, $CDCl_3$) δ 163.0, 161.4, 148.1, 135.3, 135.3, 129.4, 129.1, 129.1, 117.9, 115.6, 115.4, 113.0, 47.8. The spectral data are in agreement with previous report[5].

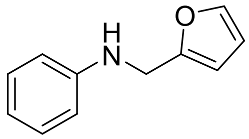


*N*-(furan-2-ylmethyl)aniline (3k)

The reaction was purified by silica gel column chromatography (petroleum ether/EtOAc 46:1). The product was isolated (6.5 mg, 30%) as a colorless oil.

$^{1}$H NMR (600 MHz, $CDCl_3$) δ 7.39 – 7.35 (m, 1H), 7.23 – 7.17 (m, 2H), 6.75 (t, *J* = 7.3 Hz, 1H), 6.71 – 6.66 (m, 2H), 6.35 – 6.31 (m, 1H), 6.25 – 6.23 (m, 1H), 4.33 (s, 2H), 4.02 (s, 1H). $^{13}C\{^{1}H\}$ NMR (151 MHz, $CDCl_3$) δ 152.9, 147.7, 142.0, 129.3, 118.1, 113.3, 110.4, 107.0, 41.6. The spectral data are in agreement with previous report[6].

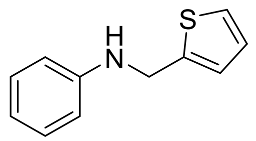


*N*-(thiophen-2-ylmethyl)aniline (3l)

The reaction was purified by silica gel column chromatography (petroleum ether/EtOAc 46:1). The product was isolated (19.8 mg, 84%) as a yellow oil.

H NMR (400 MHz, $CDCl_3$) δ 7.28 – 7.18 (m, 3H), 7.04 (s, 1H), 6.98 (t, *J* = 4.2 Hz, 1H), 6.80 (t, *J* = 7.3 Hz, 1H), 6.73 (d, *J* = 7.9 Hz, 2H), 4.53 (s, 2H). $^{13}C\{^{1}H\}$ NMR (101 MHz, $CDCl_3$) δ 147.0, 142.3, 129.4, 127.0, 125.4, 124.8, 118.7, 113.8, 43.9. The spectral data are in agreement with previous report[7].

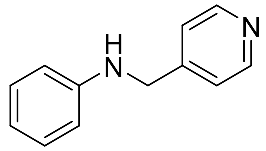


*N*-((pyridin-4-yl)methyl)benzenamine (3m)

The reaction was purified by silica gel column chromatography (petroleum ether/EtOAc 46:1). The product was isolated (18.9 mg, 82%) as a white solid[8].

$^{1}H$ NMR (400 MHz, $CDCl_3$) δ 8.56 (d, $J$ = 5.0 Hz, 2H), 7.31 (d, $J$ = 4.9 Hz, 2H), 7.17 (t, $J$ = 7.8 Hz, 2H), 6.74 (t, $J$ = 7.4 Hz, 1H), 6.58 (d, $J$ = 7.9 Hz, 2H), 4.39 (s, 2H). $^{13}C\{^{1}H\}$ NMR (101 MHz, $CDCl_3$) δ 149.8, 149.3, 147.5, 129.4, 122.2, 118.2, 113.0, 47.2.

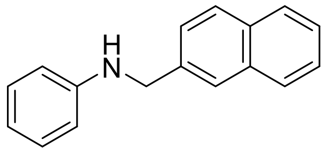

*N*-(naphthalen-2-ylmethyl)aniline (3n)

The reaction was purified by silica gel column chromatography (petroleum ether/EtOAc 46:1). The product was isolated (28.8 mg, 99%) as a yellow solid[9].

$^{1}H$ NMR (400 MHz, $CDCl_3$) δ 7.85-7.78 (m, 4H), 7.53 – 7.43 (m, 3H), 7.19 (t, $J$ = 7.8 Hz, 2H), 6.83 – 6.71 (m, 3H), 4.50 (s, 2H). $^{13}C\{^{1}H\}$ NMR (101 MHz, $CDCl_3$) δ 147.7, 136.6, 133.5, 132.9, 129.4, 128.4, 127.8, 127.8, 126.2, 126.2, 125.9, 125.8, 118.2, 113.4, 48.9.

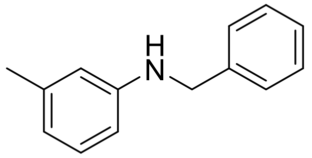

*N*-benzyl-3-methylaniline (4a)

The reaction was purified by silica gel column chromatography (petroleum ether/EtOAc 46:1). The product was isolated (21.2 mg, 86%) as a colorless oil[10].

$^{1}H$ NMR (400 MHz, $CDCl_3$) δ 7.44 – 7.25 (m, 5H), 7.10 (t, *J* = 7.7 Hz, 1H), 6.58 (d, *J* = 7.4 Hz, 1H), 6.49 (d, *J* = 13.8 Hz, 2H), 4.34 (s, 2H), 4.05 (s, 1H), 2.30 (s, 3H). $^{13}C\{^{1}H\}$ NMR (101 MHz, $CDCl_3$) δ 148.2, 139.6, 139.1, 129.2, 128.7, 127.6, 127.3, 118.7, 113.8, 110.1, 48.5, 21.7.

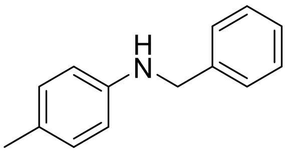


*N*-benzyl-4-methylaniline (4b)

The reaction was purified by silica gel column chromatography (petroleum ether/EtOAc 46:1). The product was isolated (21.4 mg, 87%) as a colorless oil[1].

$^{1}H$ NMR (400 MHz, $CDCl_3$) δ 7.40 – 7.22 (m, 5H), 6.98 (d, *J* = 7.7 Hz, 2H), 6.56 (d, *J* = 7.6 Hz, 2H), 4.30 (s, 2H), 4.04 (s, 1H), 2.23 (s, 3H). $^{13}C\{^{1}H\}$ NMR (101 MHz, $CDCl_3$) δ 145.9, 139.7, 129.8, 128.7, 127.6, 127.2, 126.9, 113.1, 48.8, 20.5.

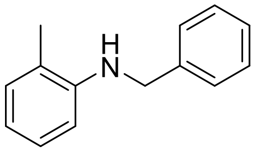


*N*-benzyl-2-methylaniline (4c)

The reaction was purified by silica gel column chromatography (petroleum ether/EtOAc 46:1). The product was isolated (19.7 mg, 80%) as a yellow oil[1].

$^{1}H$ NMR (600 MHz, $CDCl_3$) δ 7.46 – 7.37 (m, 4H), 7.36 – 7.30 (m, 1H), 7.18 – 7.09 (m, 2H), 6.73 (q, *J* = 6.8 Hz, 1H), 6.69 – 6.64 (m, 1H), 4.42 (s, 2H), 3.91 (s, 1H), 2.22 (s, 3H). $^{13}C\{^{1}H\}$

NMR (151 MHz, $CDCl_3$) δ 146.2, 139.6, 130.2, 128.7, 127.6, 127.3, 127.2, 122.0, 117.3, 110.1, 48.4, 17.6.

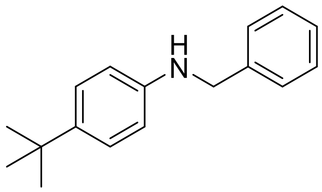

*N*-benzyl-4-tert-butylaniline (4d)

The reaction was purified by silica gel column chromatography (petroleum ether/EtOAc 46:1). The product was isolated (28.1 mg, 94%) as a yellow oil[11].

$^1H$ NMR (400 MHz, $CDCl_3$) δ 7.45 – 7.25 (m, 4H), 7.25 – 7.18 (m, 1H), 7.22 (dd, *J* = 8.7, 2.2 Hz, 2H), 6.61 (d, *J* = 8.6 Hz, 2H), 4.32 (s, 2H), 3.94 (s, 1H), 1.29 (s, 9H). $^{13}C\{^1H\}$ NMR (101 MHz, $CDCl_3$) δ 145.9, 140.4, 139.8, 128.7, 127.6, 127.2, 126.1, 112.6, 48.7, 33.9, 31.6.

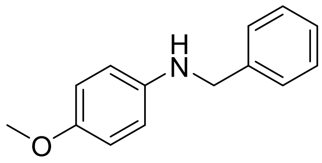

*N*-benzyl-4-methoxyaniline (4e)

The reaction was purified by silica gel column chromatography (petroleum ether/EtOAc 46:1). The product was isolated (22.6 mg, 94%) as a yellow oil[11].

$^1H$ NMR (400 MHz, $CDCl_3$) δ 7.42 – 7.22 (m, 5H), 6.79 (d, *J* = 8.7 Hz, 2H), 6.63 (d, *J* = 8.6 Hz, 2H), 4.30 (s, 2H), 3.75 (s, 3H). $^{13}C\{^1H\}$ NMR (101 MHz, $CDCl_3$) δ 152.4, 142.3, 128.7, 127.7, 127.3, 115.0, 114.4, 55.9, 49.5.

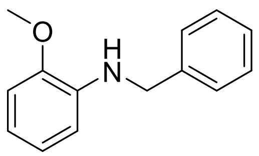

*N*-benzyl-2-methoxyaniline (4f)

The reaction was purified by silica gel column chromatography (petroleum ether/EtOAc 46:1). The product was isolated (24.0 mg, 90%) as a yellow oil[12].

$^{1}$H NMR (400 MHz, $CDCl_3$) δ 7.52 – 7.24 (m, 5H), 6.93 – 6.79 (m, 2H), 6.71 (t, *J* = 7.7 Hz, 1H), 6.63 (d, *J* = 6.0 Hz, 1H), 4.67 (s, 1H), 4.38 (s, 2H), 3.87 (s, 3H). $^{13}$C{$^{1}$H} NMR (101 MHz, $CDCl_3$) δ 146.8, 139.6, 138.2, 128.6, 127.6, 127.2, 121.3, 116.7, 110.1, 109.5, 55.5, 48.1.

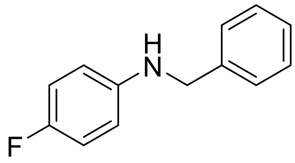

*N*-benzyl-4-fluoroaniline (4g)

The reaction was purified by silica gel column chromatography (petroleum ether/EtOAc 98:2). The product was isolated (20.9 mg, 83%) as a yellow oil[9].

$^{1}$H NMR (600 MHz, $CDCl_3$) δ 7.41 – 7.34 (m, 4H), 7.33 – 7.27 (m, 1H), 6.90 (t, *J* = 8.7 Hz, 2H), 6.61 – 6.55 (m, 2H), 4.31 (s, 2H), 3.94 (s, 1H). $^{13}$C{$^{1}$H} NMR (151 MHz, $CDCl_3$) δ 156.8, 155.2, 144.6, 139.4, 128.7, 127.6, 127.4, 115.8, 115.7, 113.8, 113.7, 49.0.

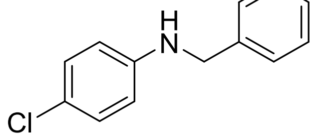

*N*-benzyl-4-chloroaniline (4h)

The reaction was purified by silica gel column chromatography (petroleum ether/EtOAc 46:1). The product was isolated (23.6 mg, 87%) as a white solid[9].

$^1$H NMR (400 MHz, $CDCl_3$) δ 7.40 – 7.27 (m, 5H), 7.16 – 7.09 (m, 2H), 6.65 – 6.56 (m, 2H), 4.31 (s, 2H). $^{13}C\{^1H\}$ NMR (101 MHz, $CDCl_3$) δ 146.5, 138.8, 129.1, 128.7, 127.5, 127.4, 122.3, 114.2, 48.5.

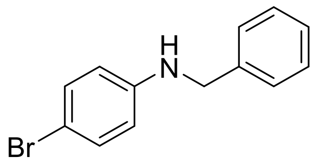


*N*-benzyl-4-bromoaniline (4i)

The reaction was purified by silica gel column chromatography (petroleum ether/EtOAc 46:1). The product was isolated (27.1 mg, 83%) as a yellow oil[9].

$^1$H NMR (600 MHz, $CDCl_3$) δ 7.38 – 7.33 (m, 4H), 7.32 – 7.27 (m, 1H), 7.26 – 7.23 (m, *2*H), 6.53 – 6.49 (m, 2H), 4.31 (s, 2H), 4.07 (s, 1H). $^{13}C\{^1H\}$ NMR (151 MHz, $CDCl_3$) δ 147.1, 138.9, 132.0, 128.8, 127.5, 127.5, 114.6, 109.3, 48.4.

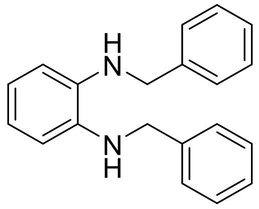


*N*, *N'*-dibenzyl-1,2-phenylenediamine (4j)

The reaction was purified by silica gel column chromatography (petroleum ether/EtOAc 46:1). The product was isolated (29.5 mg, 82%) as a yellow oil[13].

$^{1}H$ NMR (400 MHz, $CDCl_3$) δ 7.69 – 7.18 (m, 10H), 6.79 (d, $J$ = 27.2 Hz, 4H), 4.34 (s, 4H), 3.66 (s, 2H). $^{13}C\{^{1}H\}$ NMR (101 MHz, $CDCl_3$) δ 139.5, 137.2, 128.7, 127.9, 127.3, 119.5, 112.1, 48.9.

# $^{1}$H and $^{13}$C NMR spectra of products

## *N*-benzylaniline (3a)

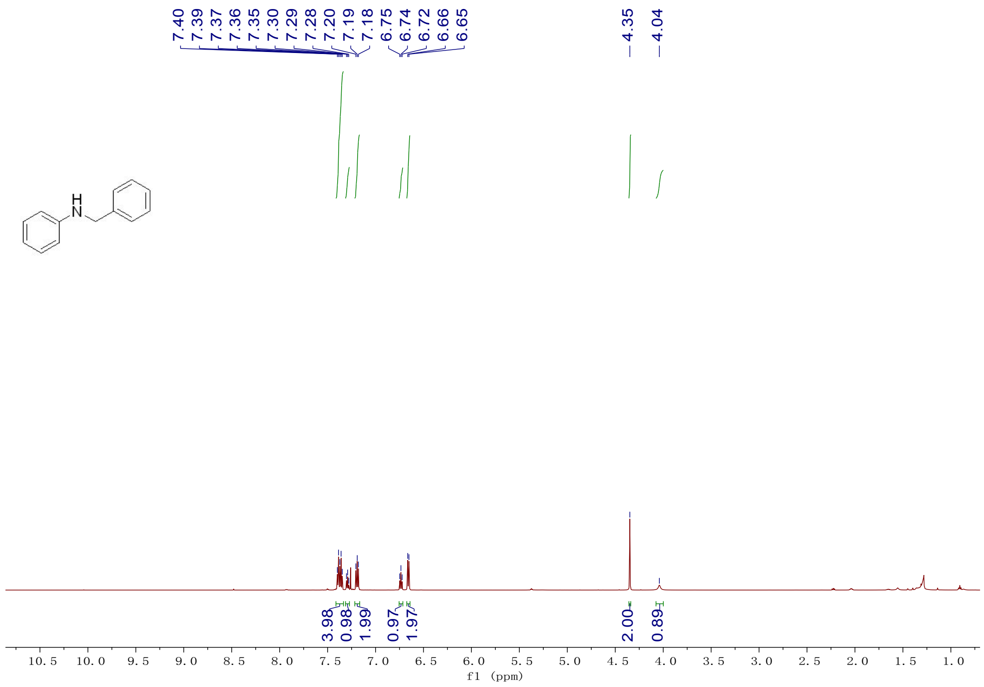


**Figure S3**. $^{1}$H NMR spectra of **3a** (600 MHz, $CDCl_3$).

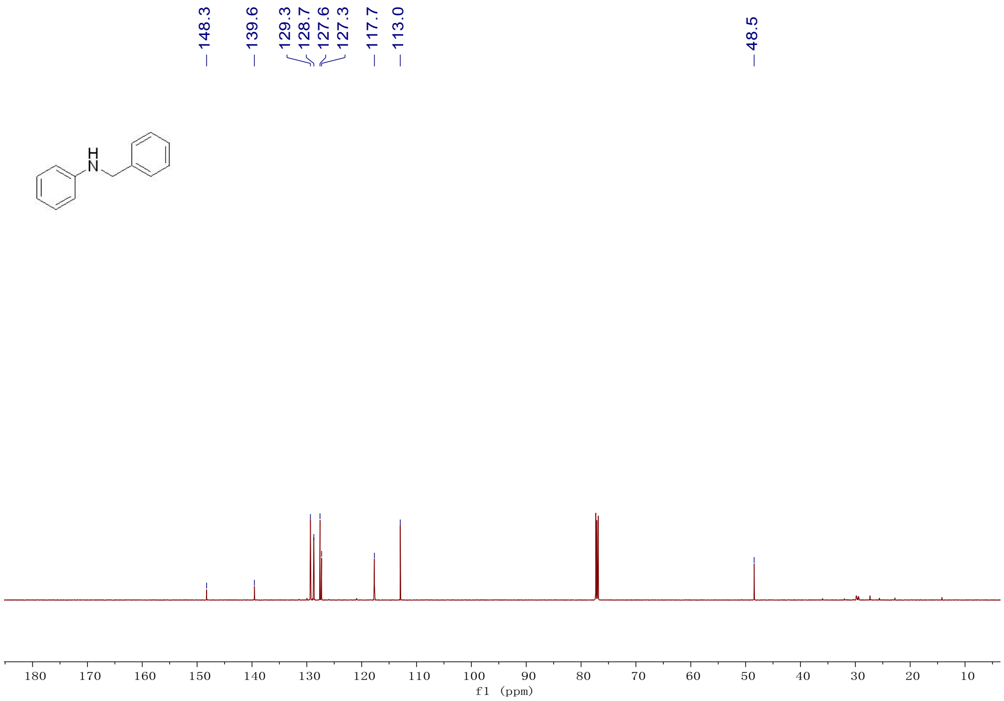

**Figure S4.** $^{13}C\{^{1}H\}$ NMR spectra of **3a** (151 MHz, $CDCl_3$).

**_N_-(2-methylbenzyl)aniline (3b)**

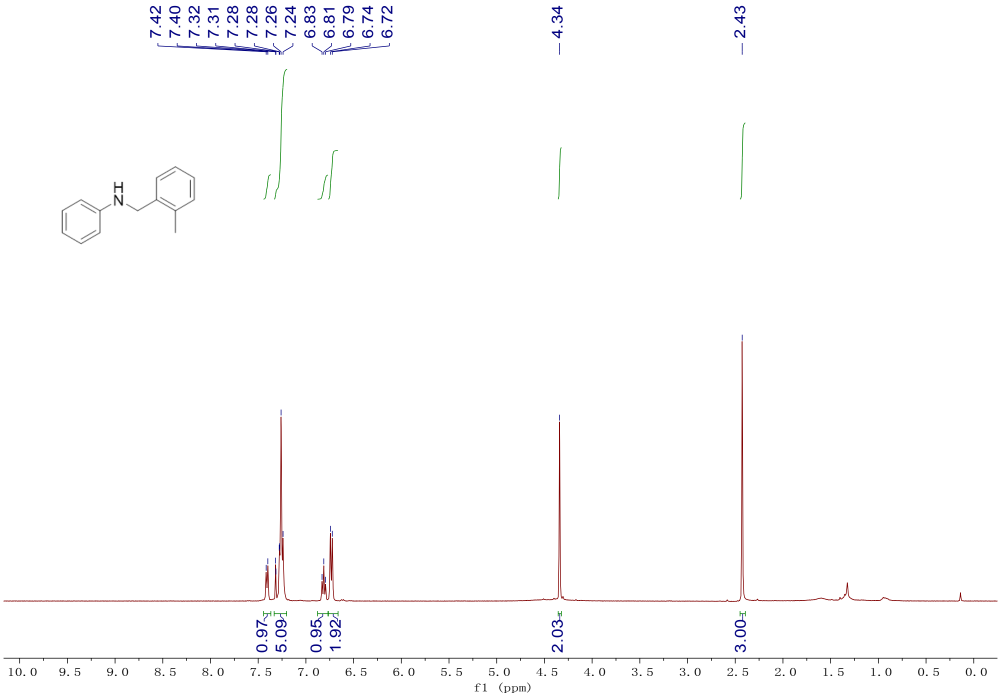


**Figure S5.** $^{1}H$ NMR spectra of **3b** (400 MHz, $CDCl_3$).

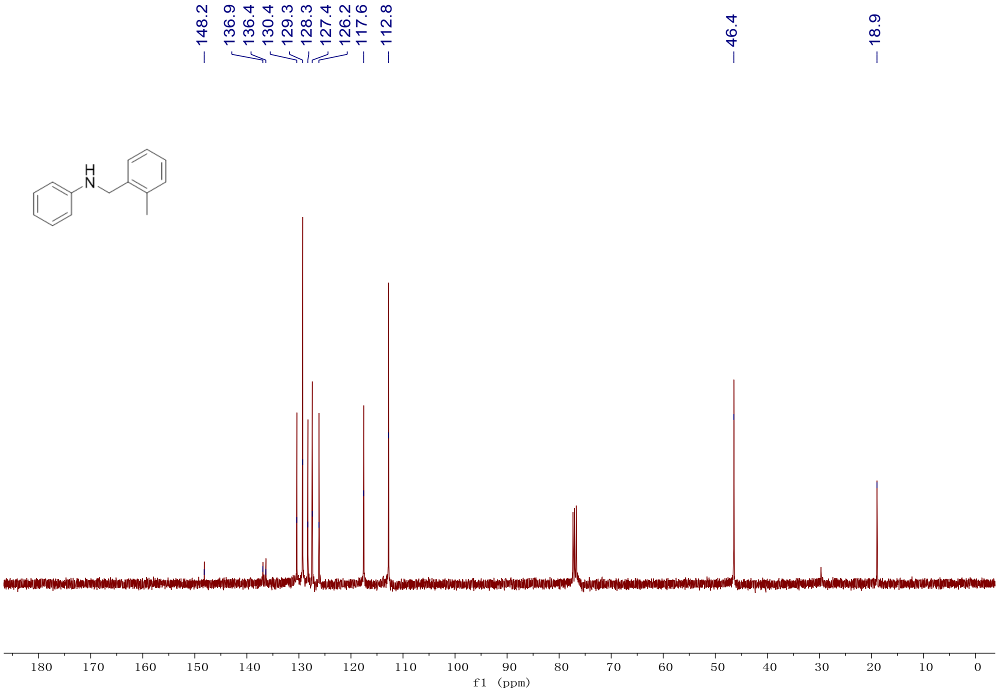


**Figure S6.** $^{13}C\{^{1}H\}$ NMR spectra of **3b** (101 MHz, $CDCl_3$).

***N*-(3-methylbenzyl)aniline (3c)**

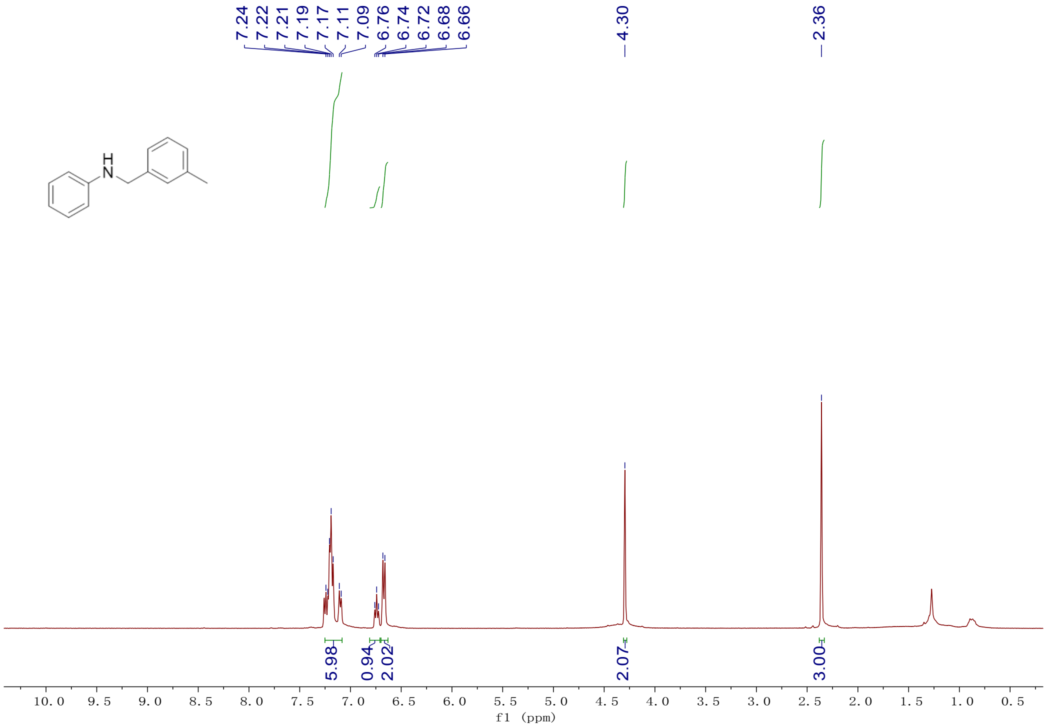


**Figure S7.** $^{1}H$ NMR spectra of **3c** (400 MHz, $CDCl_3$).

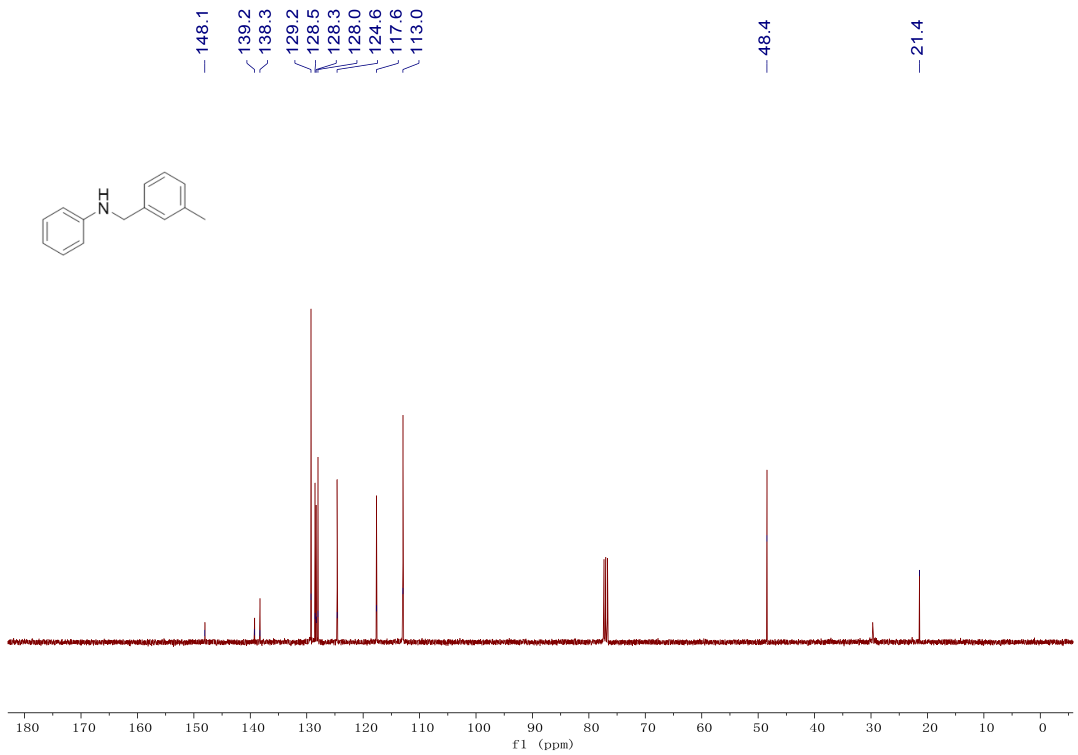


**Figure S8.** $^{13}C\{^1H\}$ NMR spectra of **3c** (101 MHz, $CDCl_3$).

***N*-(4-methylbenzyl)aniline (3d)**

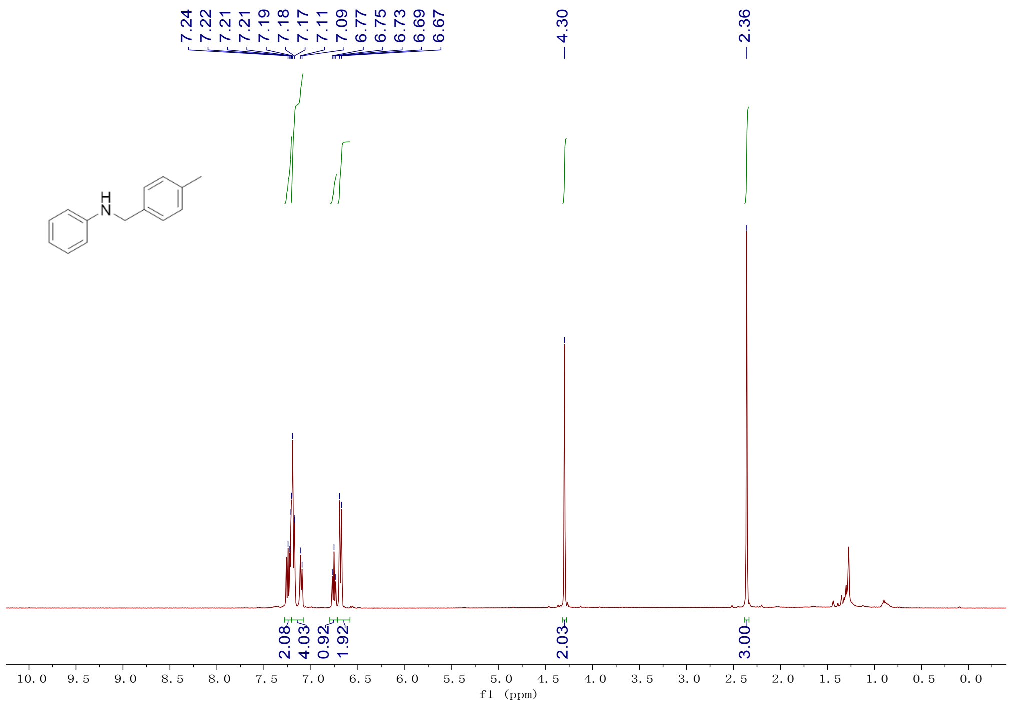


**Figure S9.** $^1H$ NMR spectra of **3d** (400 MHz, $CDCl_3$).

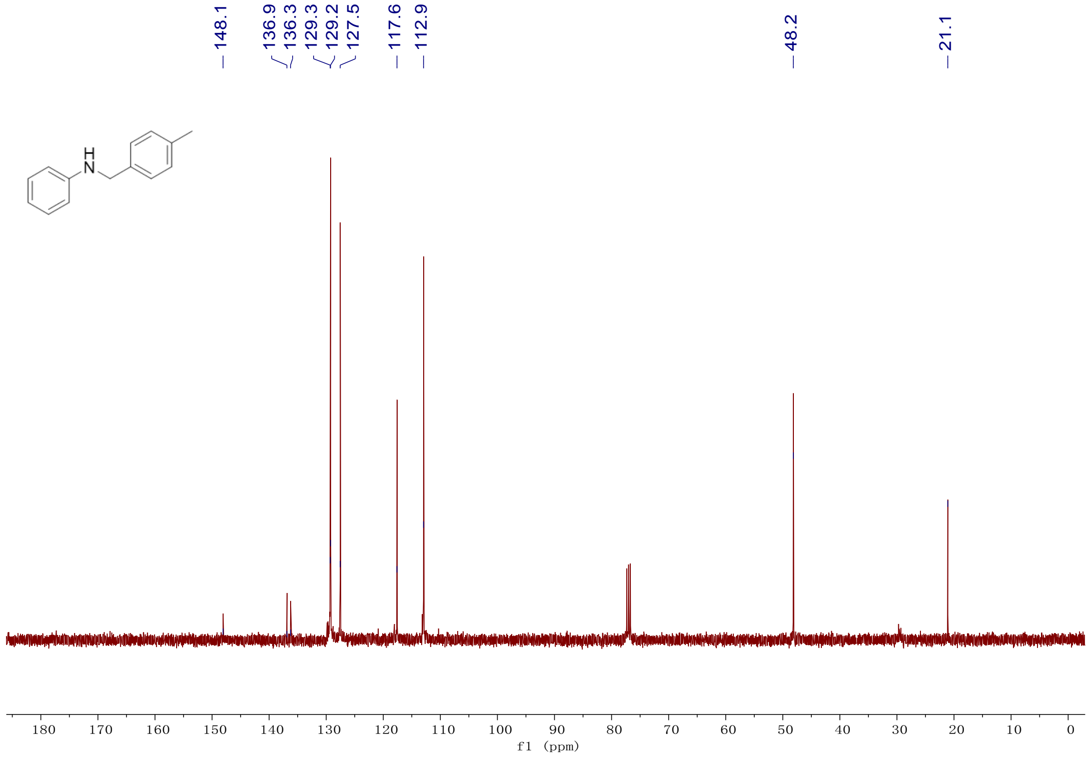


**Figure S10.** $^{13}C\{^{1}H\}$ NMR spectra of **3d** (101 MHz, $CDCl_3$).

**_N_-(4-Isopropylbenzyl)aniline (3e)**

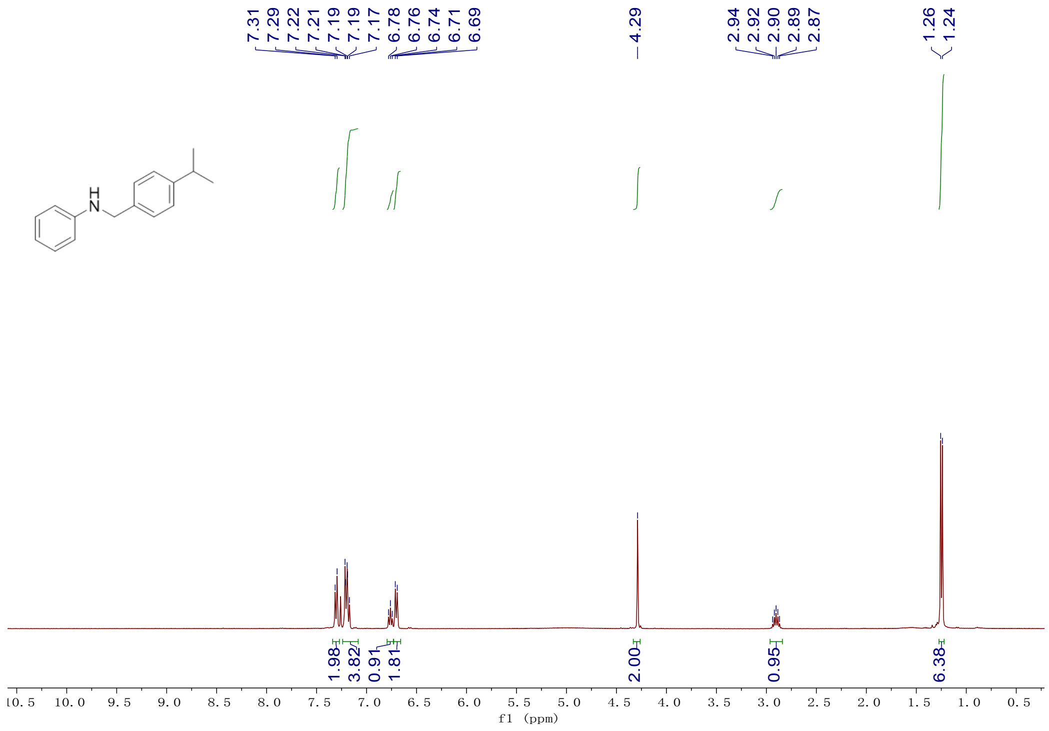

**Figure S11.** $^{1}H$ NMR spectra of **3e** (400 MHz, $CDCl_3$).

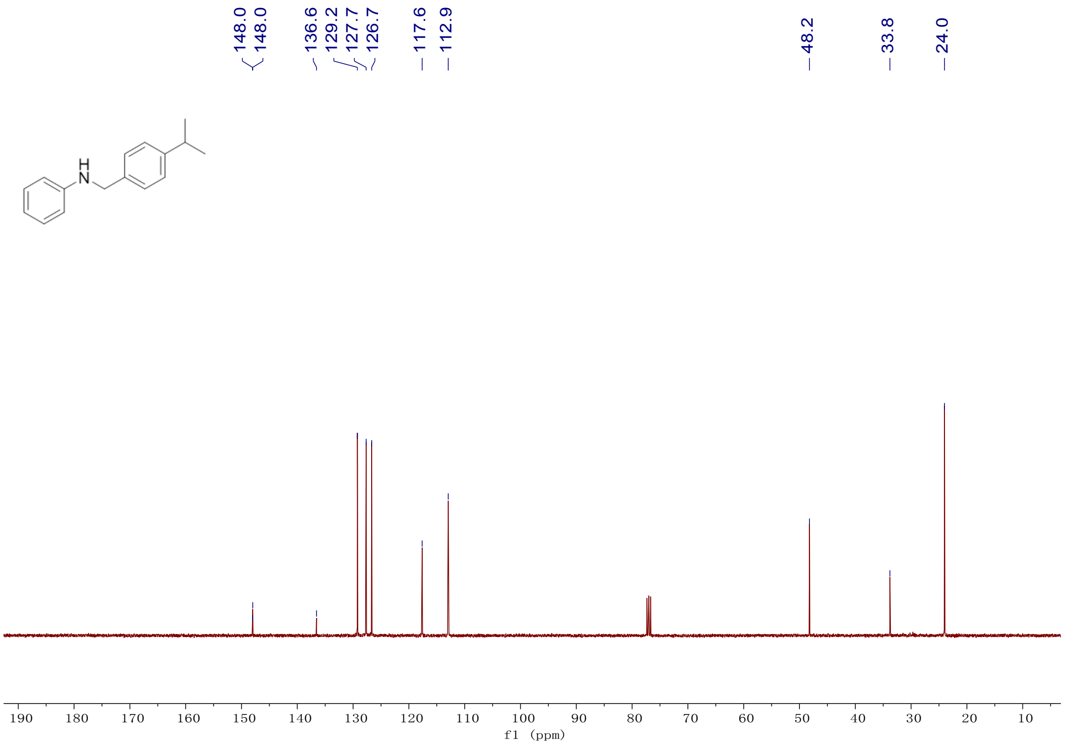


**Figure S12.** $^{13}C\{^{1}H\}$ NMR spectra of **3e** (101 MHz, $CDCl_3$).

***N*-(3-methoxybenzyl)aniline (3f)**

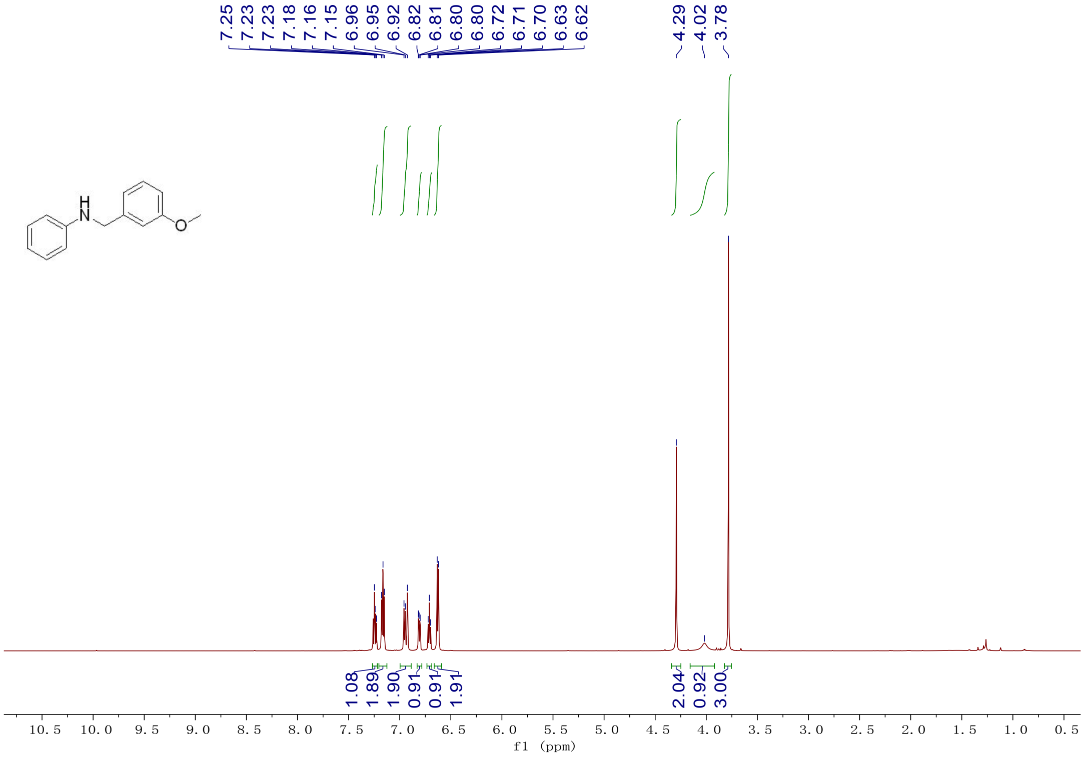

**Figure S13.** $^{1}H$ NMR spectra of **3f** (600 MHz, $CDCl_3$).

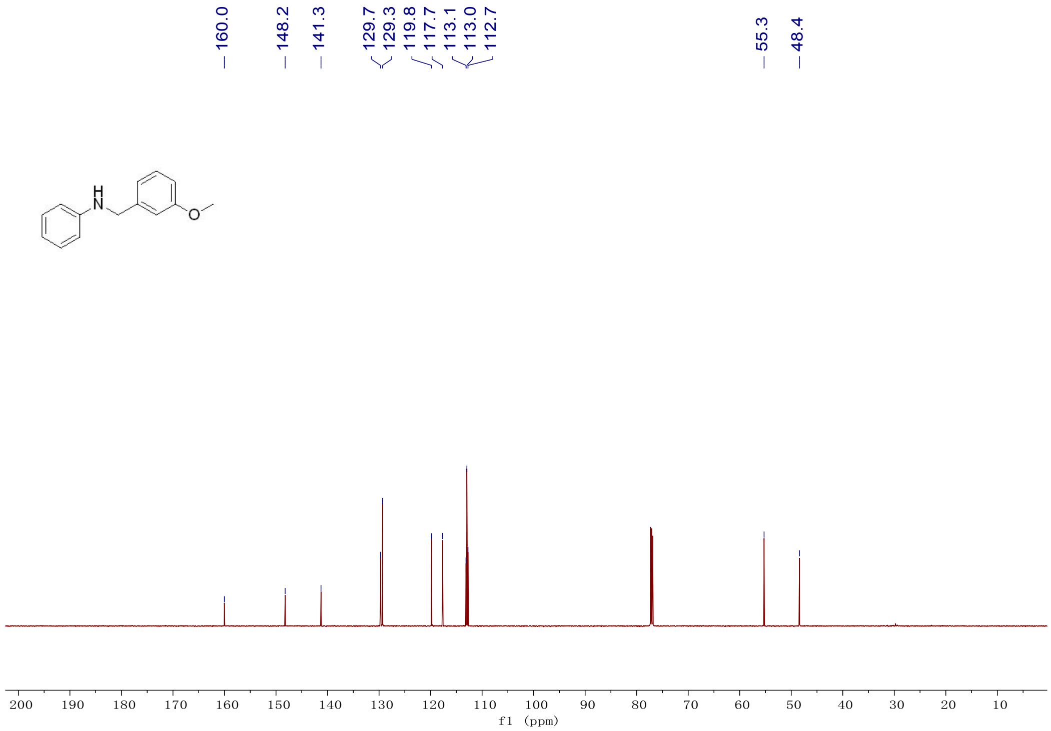

**Figure S14.** $^{13}C\{^{1}H\}$ NMR spectra of **3f** (151 MHz, $CDCl_3$).

***N*-((4-methoxy)benzyl)aniline (3g)**

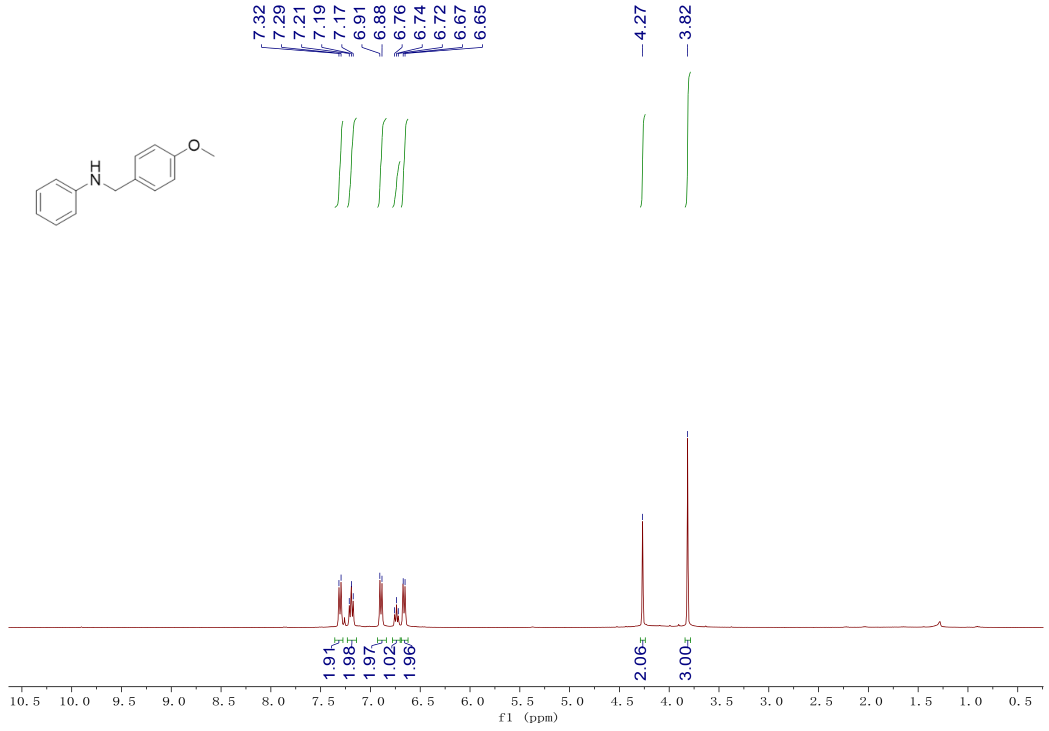


**Figure S15.** $^{1}$H NMR spectra of **3g** (400 MHz, $CDCl_3$).

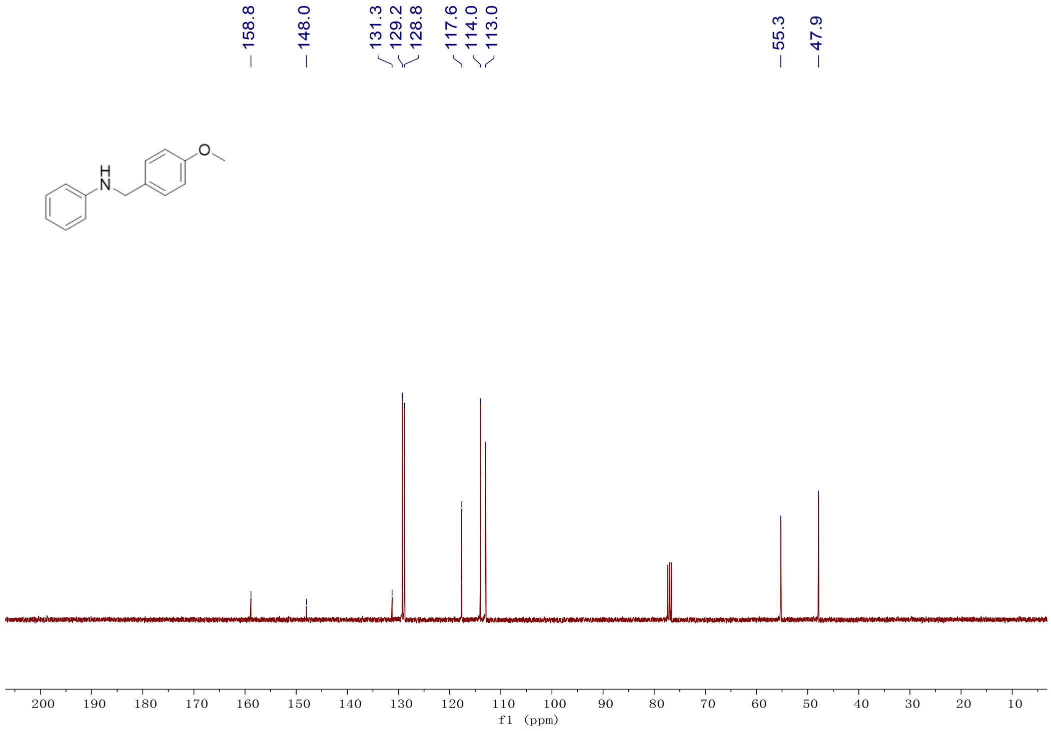


**Figure S16.** $^{13}C\{^{1}H\}$ NMR spectra of **3g** (101 MHz, $CDCl_3$).

## *N*-(4-bromobenzyl)aniline (3h)

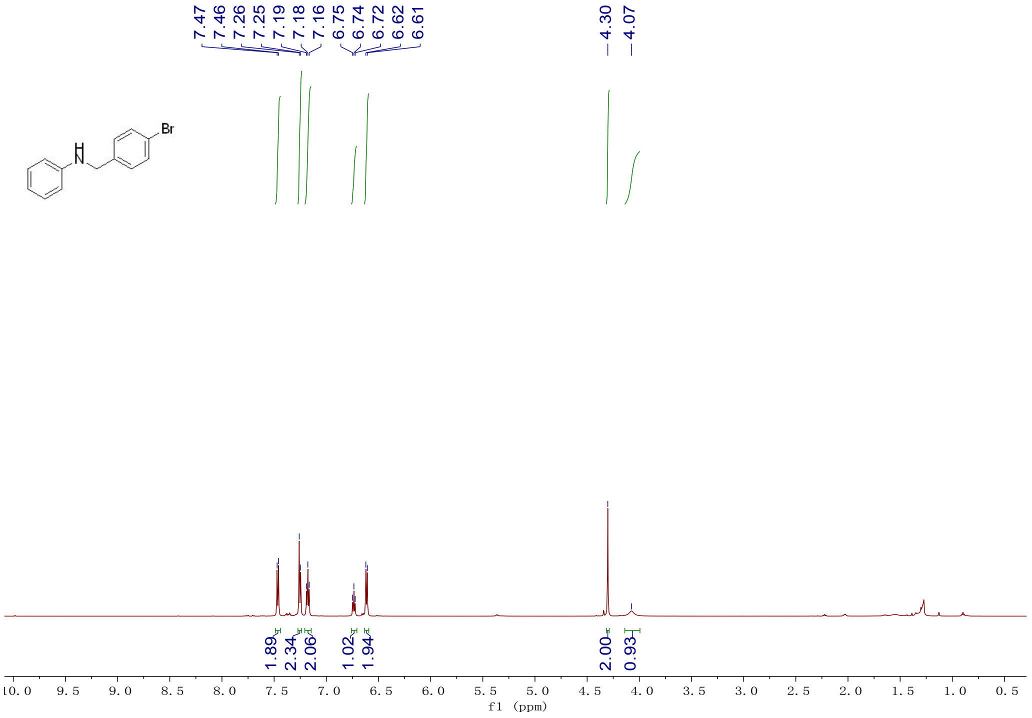


**Figure S17.** $^{13}$C NMR spectra of **3h** (600 MHz, $CDCl_3$).

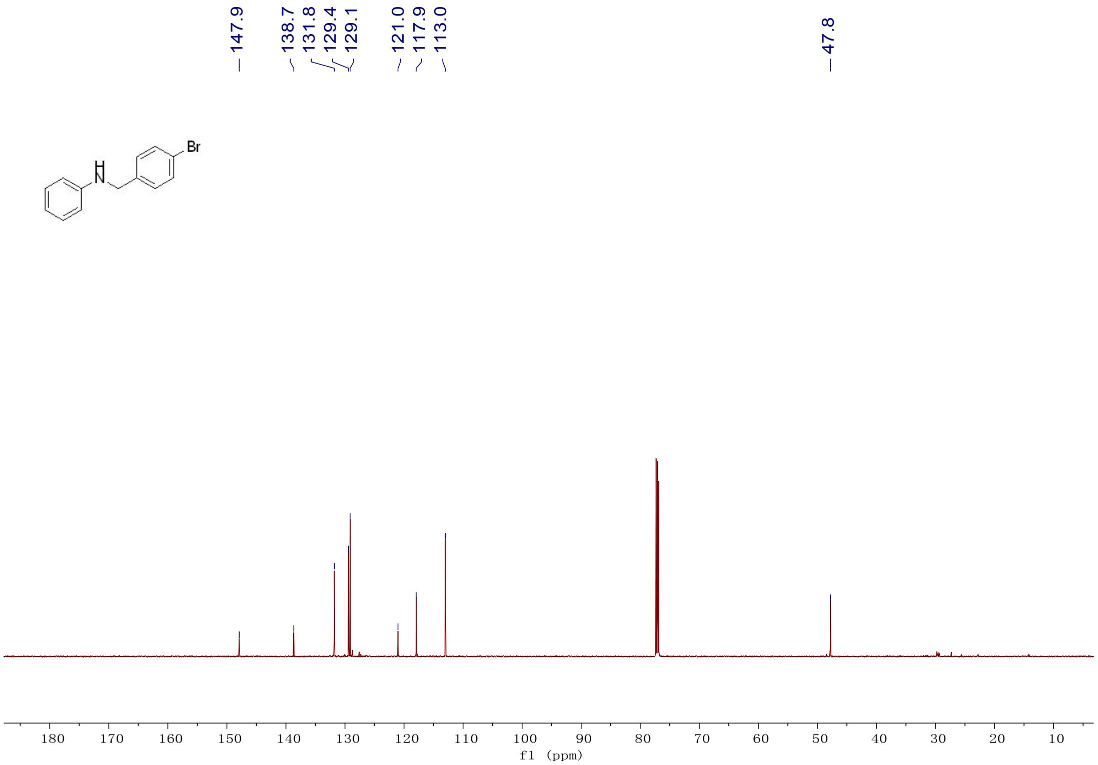

**Figure S18.** $^{13}C\{^1H\}$ NMR spectra of **3h** (151 MHz, $CDCl_3$).

***N*-(4-chlorobenzyl)aniline (3i)**

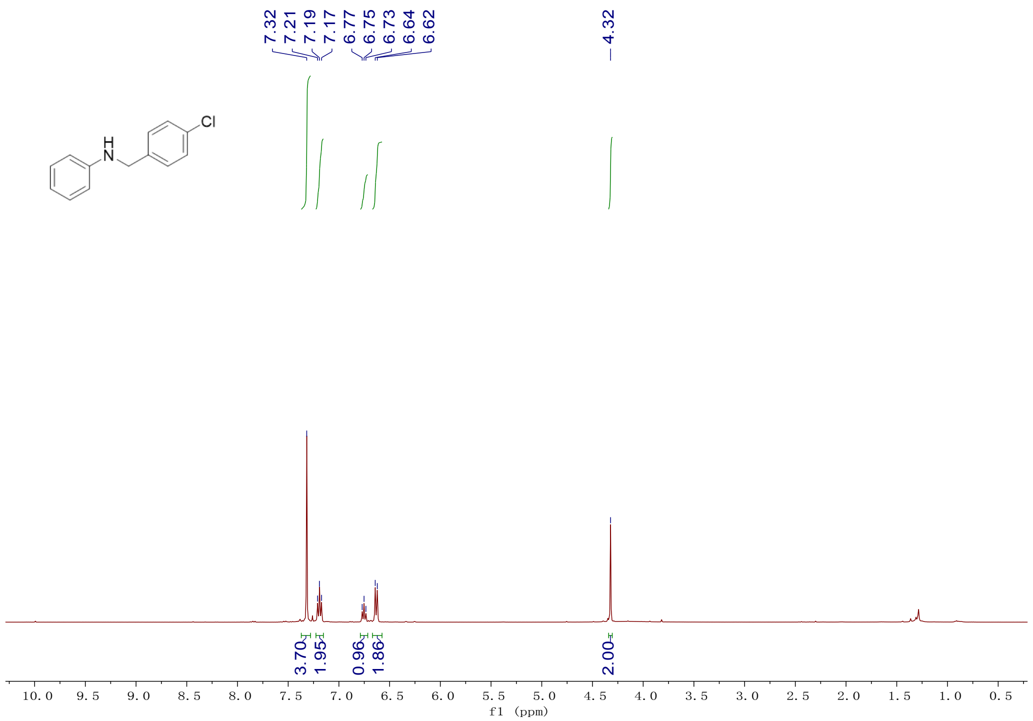

**Figure S19.** $^1H$ NMR spectra of **3i** (400 MHz, $CDCl_3$).

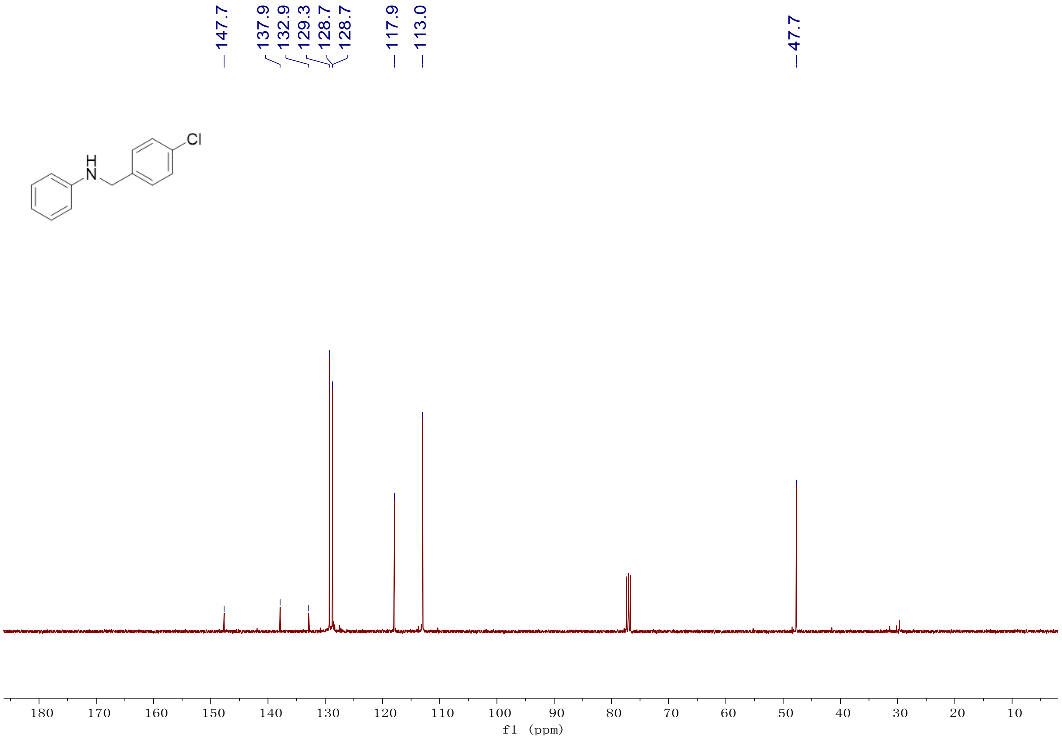

**Figure S20.** $^{13}C\{^{1}H\}$ NMR spectra of **3i** (101 MHz, $CDCl_3$).

***N*-(4-fluorobenzyl)aniline (3j)**

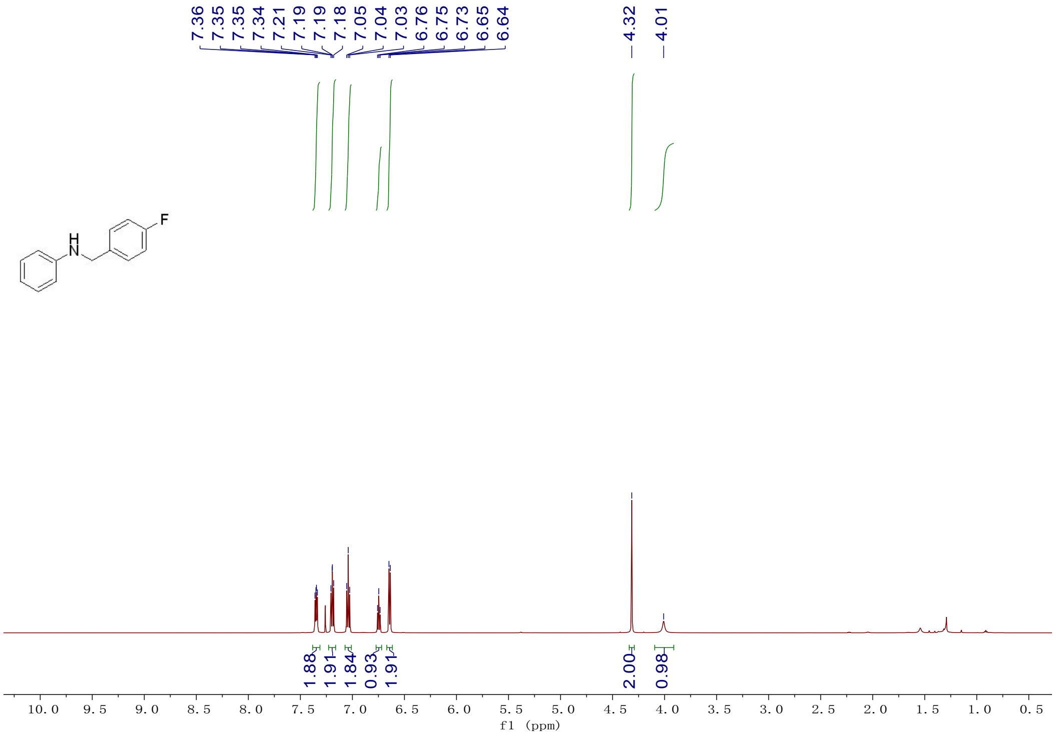


**Figure S21.** $^{1}H$ NMR spectra of **3j** (600 MHz, $CDCl_3$).

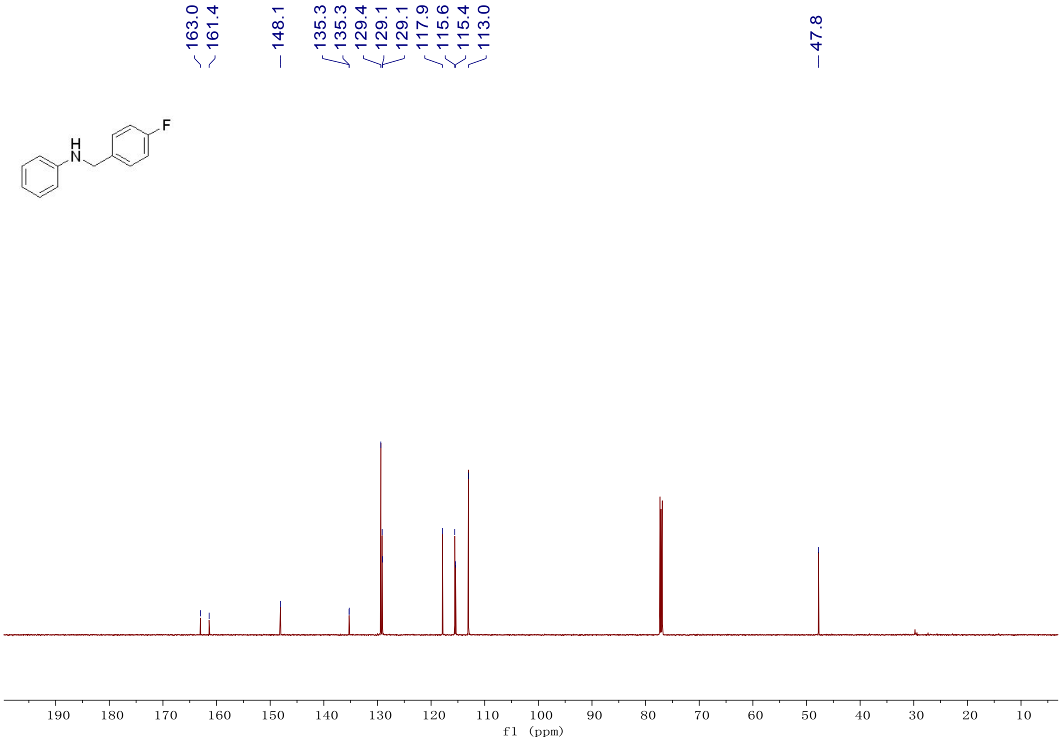

**Figure S22.** $^{13}C\{^{1}H\}$ NMR spectra of **3j** (151 MHz, $CDCl_3$).

***N*-(furan-2-ylmethyl)aniline (3k)**

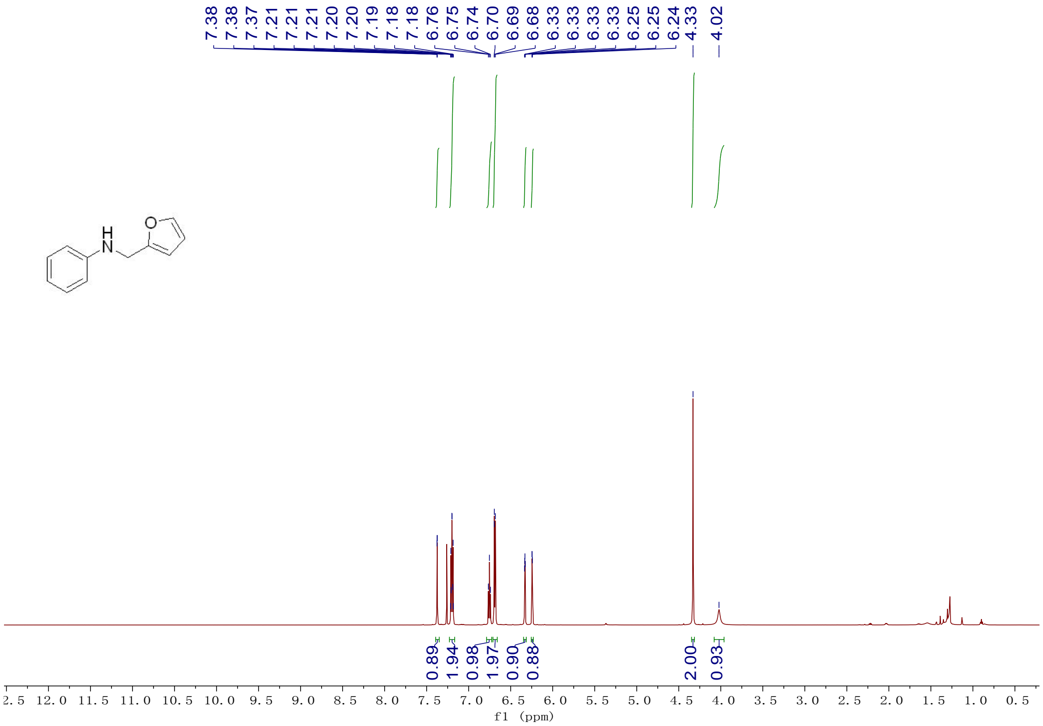


**Figure S23.** $^{1}H$ NMR spectra of **3k** (600 MHz, $CDCl_3$).

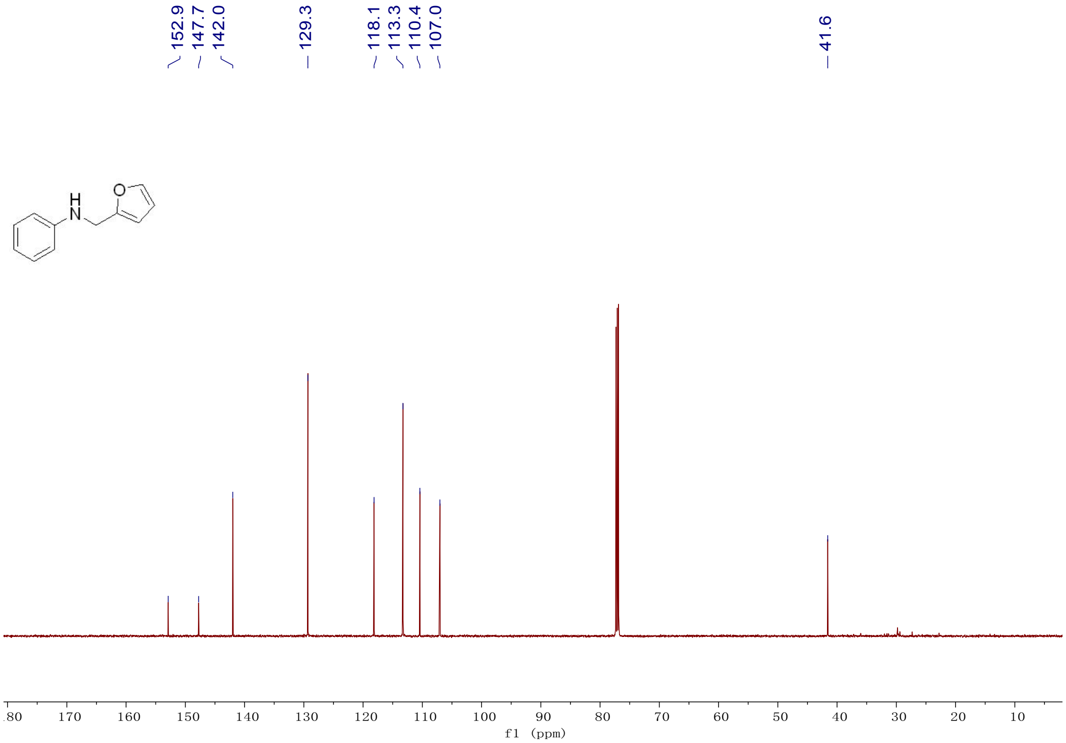

**Figure S24.** $^{13}C\{^{1}H\}$ NMR spectra of **3k** (151 MHz, $CDCl_3$).

***N*-(thiophen-2-ylmethyl)aniline (3l)**

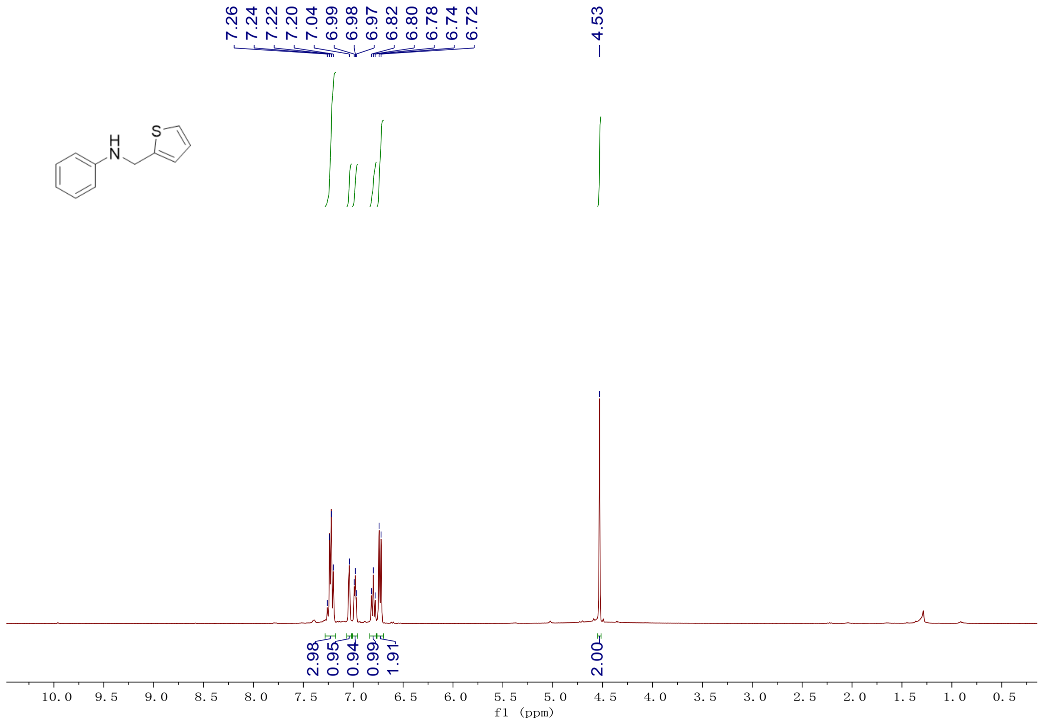


**Figure S25.** $^{1}H$ NMR spectra of **3l** (400 MHz, $CDCl_3$).

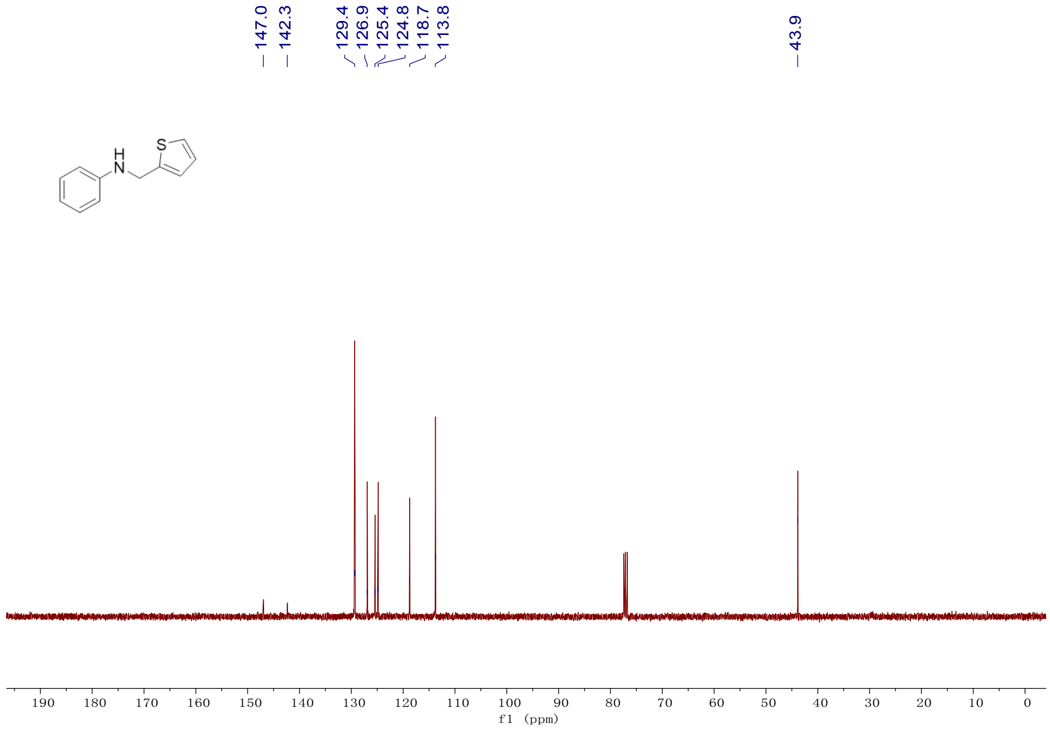

**Figure S26.** $^{13}C\{^{1}H\}$ NMR spectra of **3l** (101 MHz, $CDCl_3$).

## ***N*-((pyridin-4-yl)methyl)benzenamine (3m)**

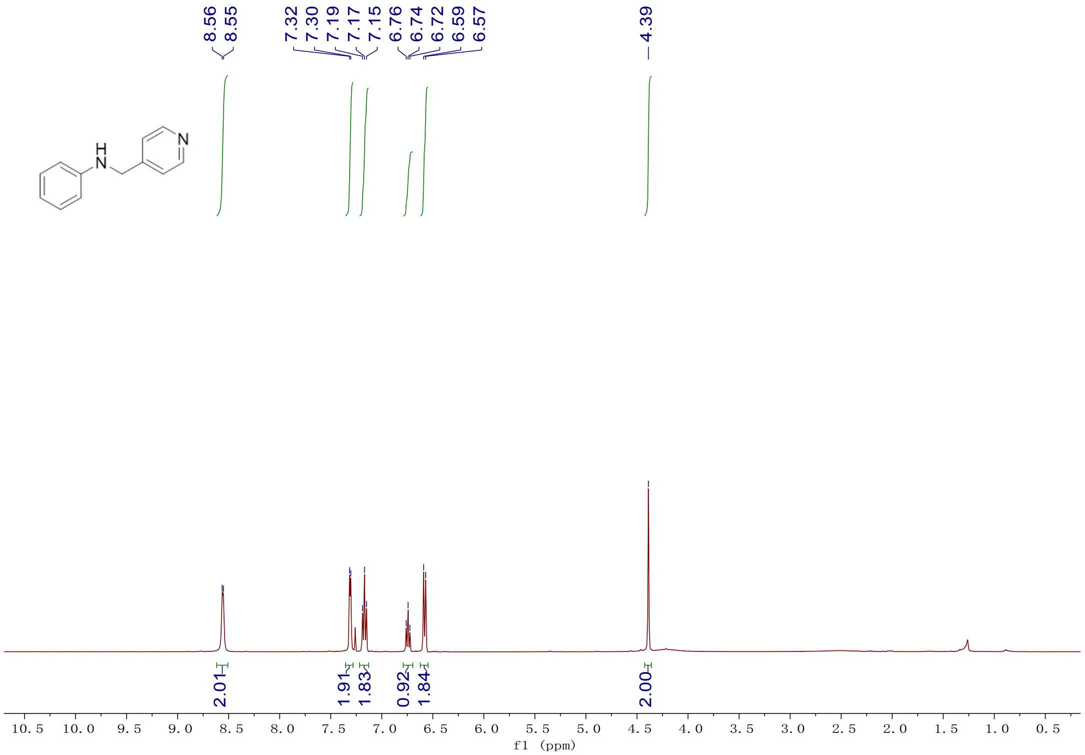


**Figure S27.** $^{1}H$ NMR spectra of **3m** (400 MHz, $CDCl_3$).

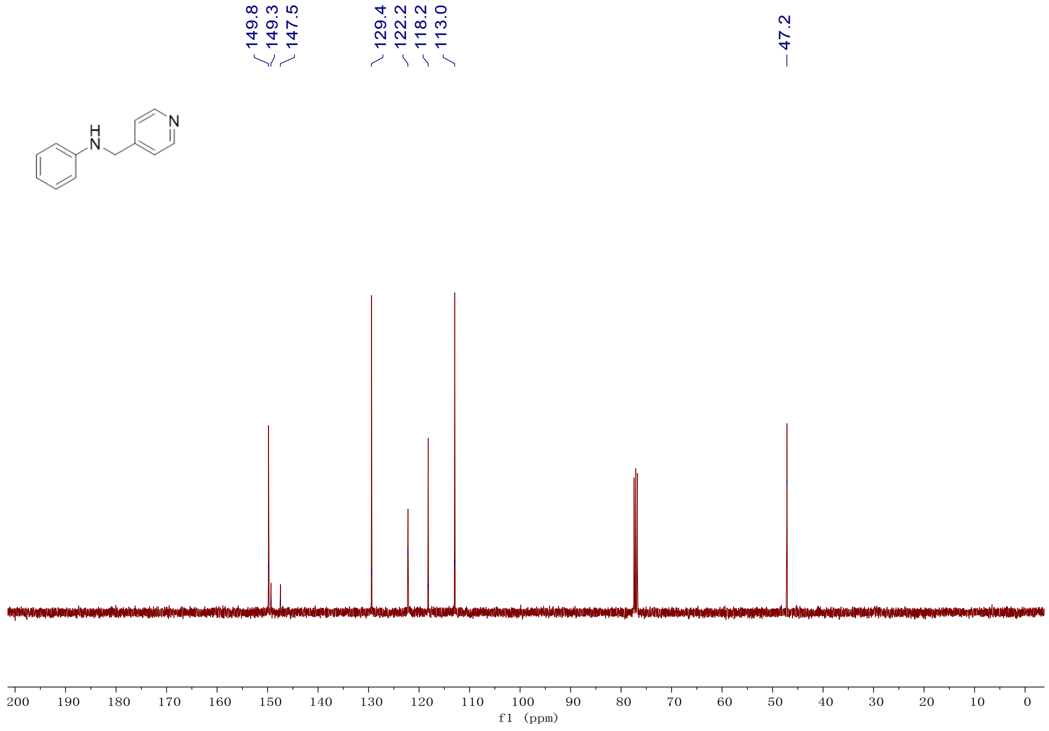

**Figure S28.** $^{13}C\{^{1}H\}$ NMR spectra of **3m** (101 MHz, $CDCl_3$).

***N*-(naphthalen-2-ylmethyl)aniline (3n)**

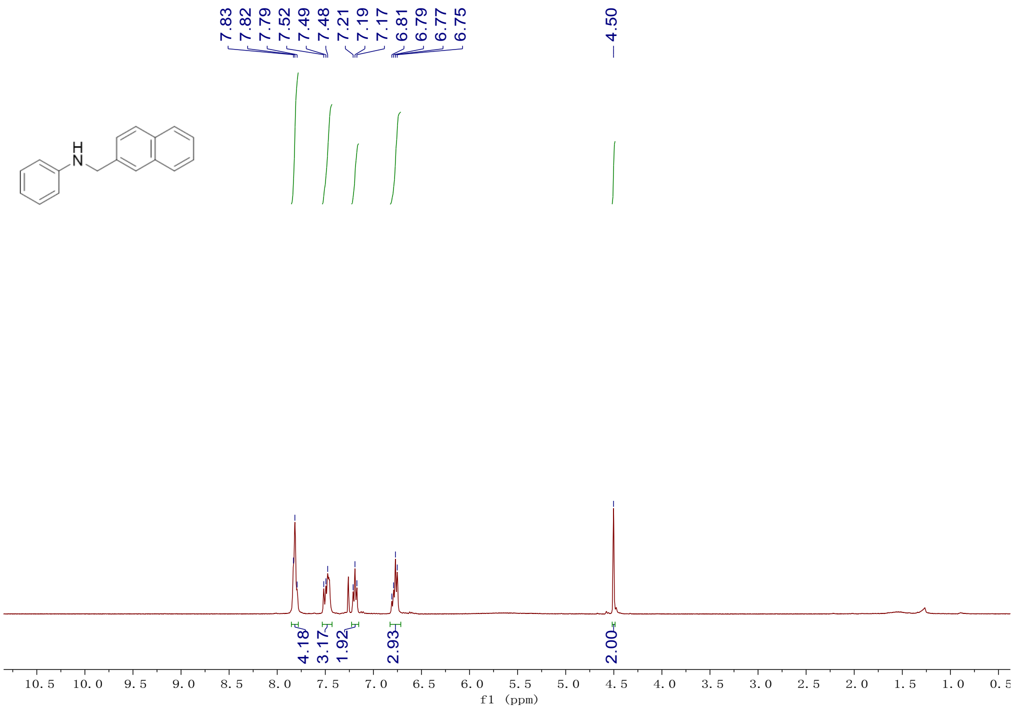


**Figure S29.** $^{1}H$ NMR spectra of **3n** (400 MHz, $CDCl_3$).

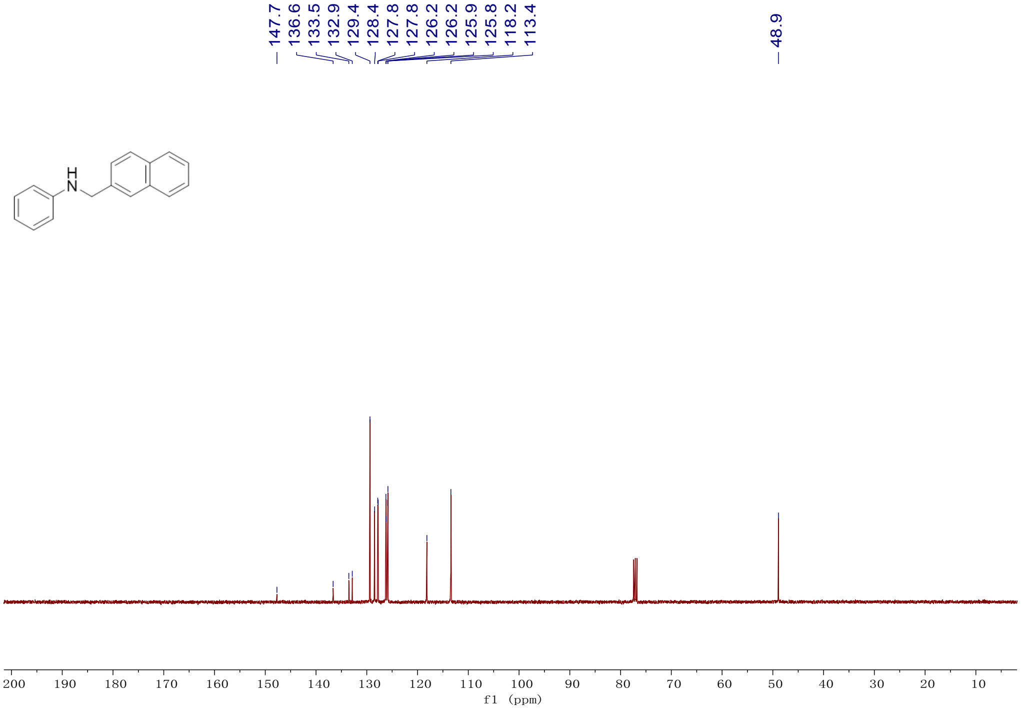

**Figure S30.** $^{13}C\{^{1}H\}$ NMR spectra of **3n** (101 MHz, $CDCl_3$).

## ***N*-benzyl-3-methylaniline (4a)**

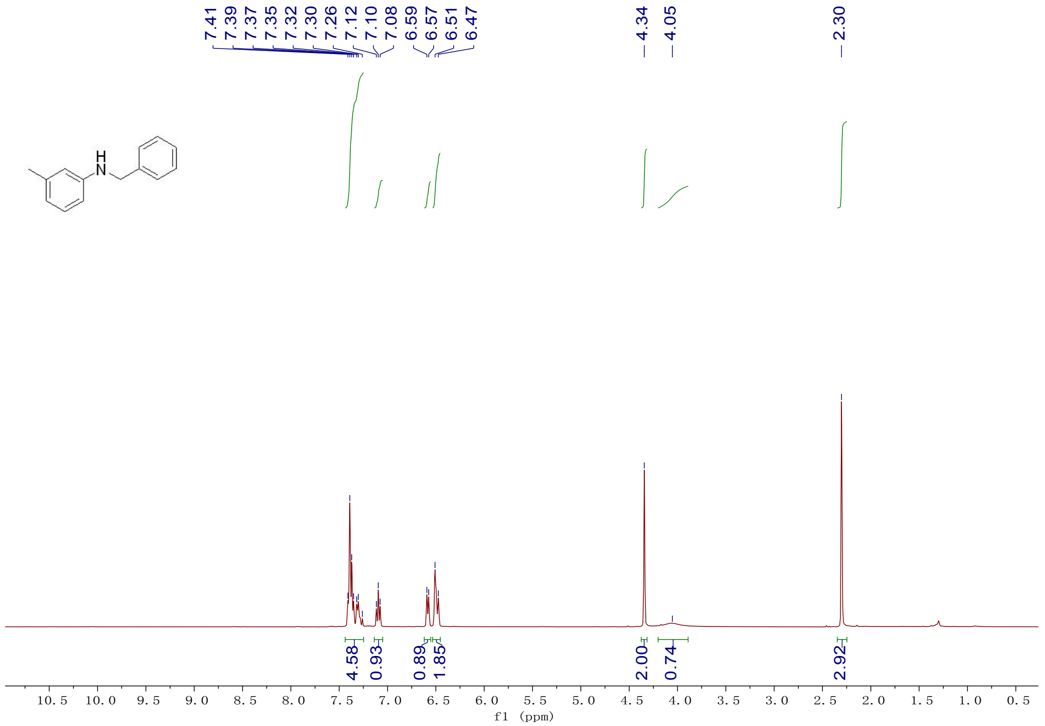


**Figure S31.** $^{1}H$ NMR spectra of **4a** (400 MHz, $CDCl_3$).

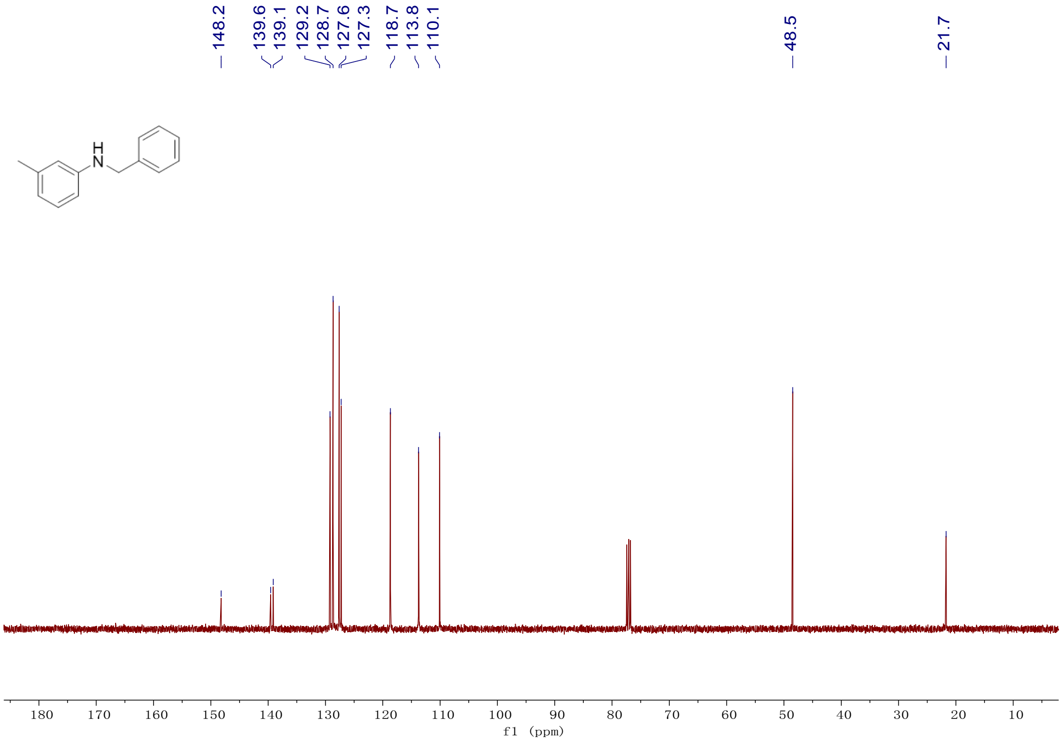

**Figure S32.** $^{13}C\{^{1}H\}$ NMR spectra of **4a** (101 MHz, $CDCl_3$).

**_N_-benzyl-4-methylaniline (4b)**

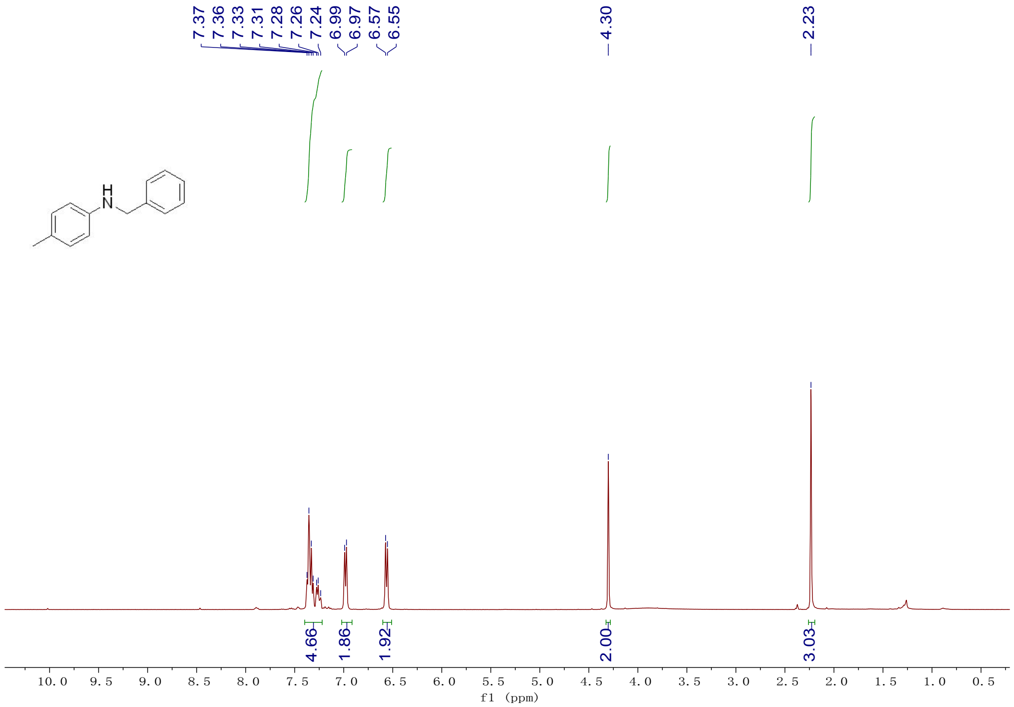


**Figure S33.** $^{1}H$ NMR spectra of **4b** (400 MHz, $CDCl_3$).

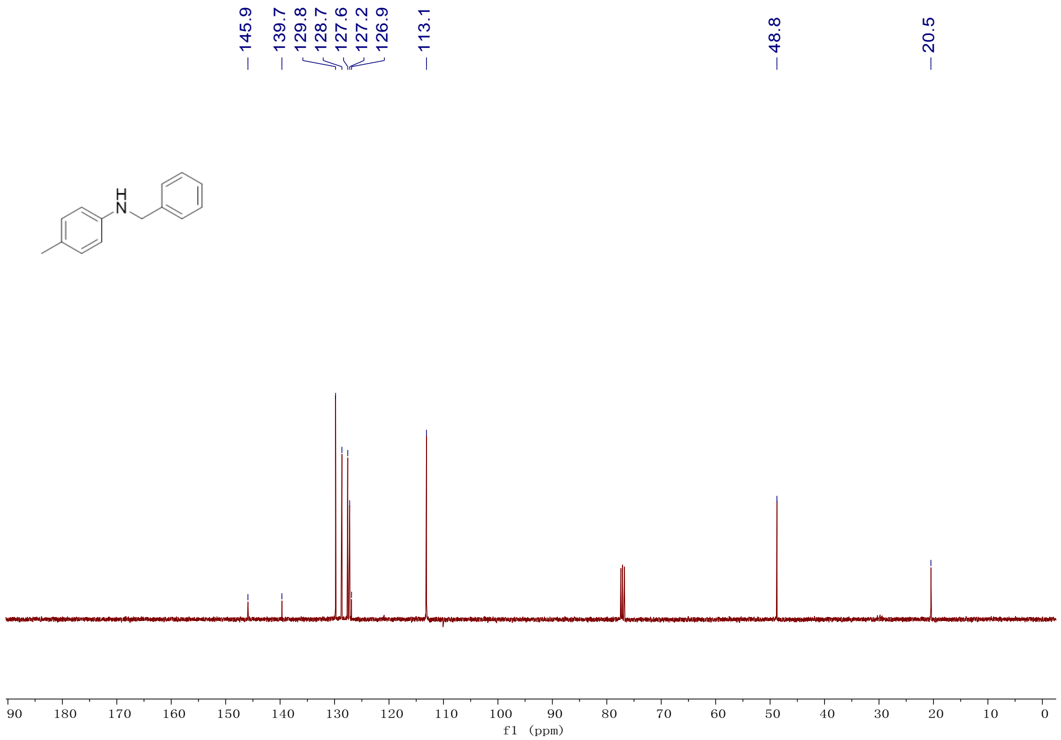

**Figure S34.** $^{13}C\{^{1}H\}$ NMR spectra of **4b** (101 MHz, $CDCl_3$).

***N*- benzyl-2-methylaniline (4c)**

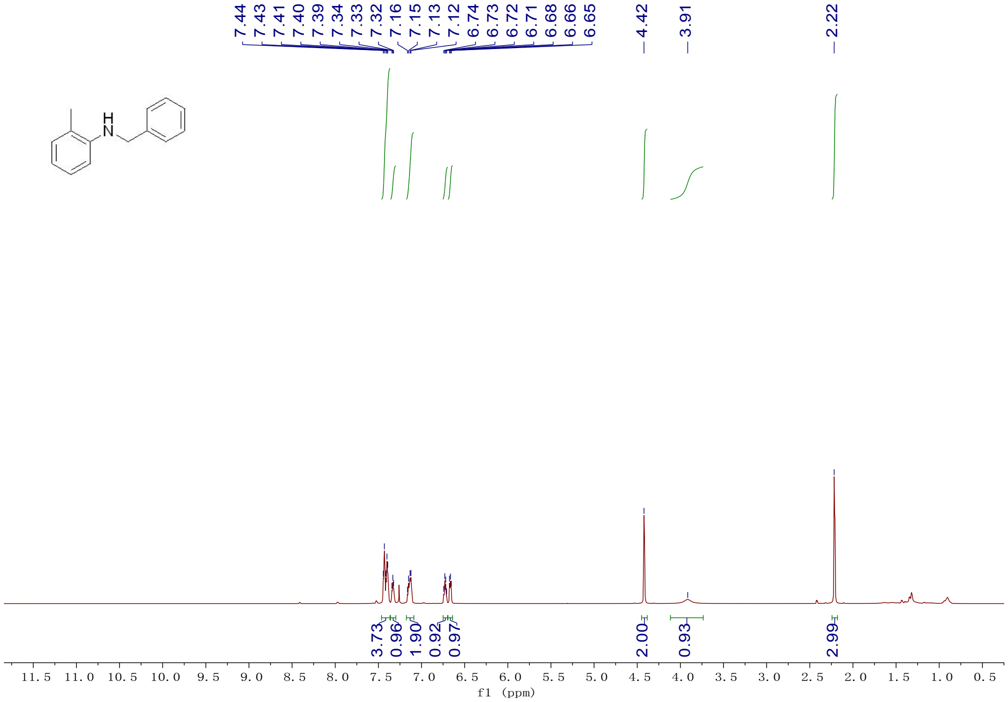


**Figure S35.** $^{1}H$ NMR spectra of **4c** (600 MHz, $CDCl_3$).

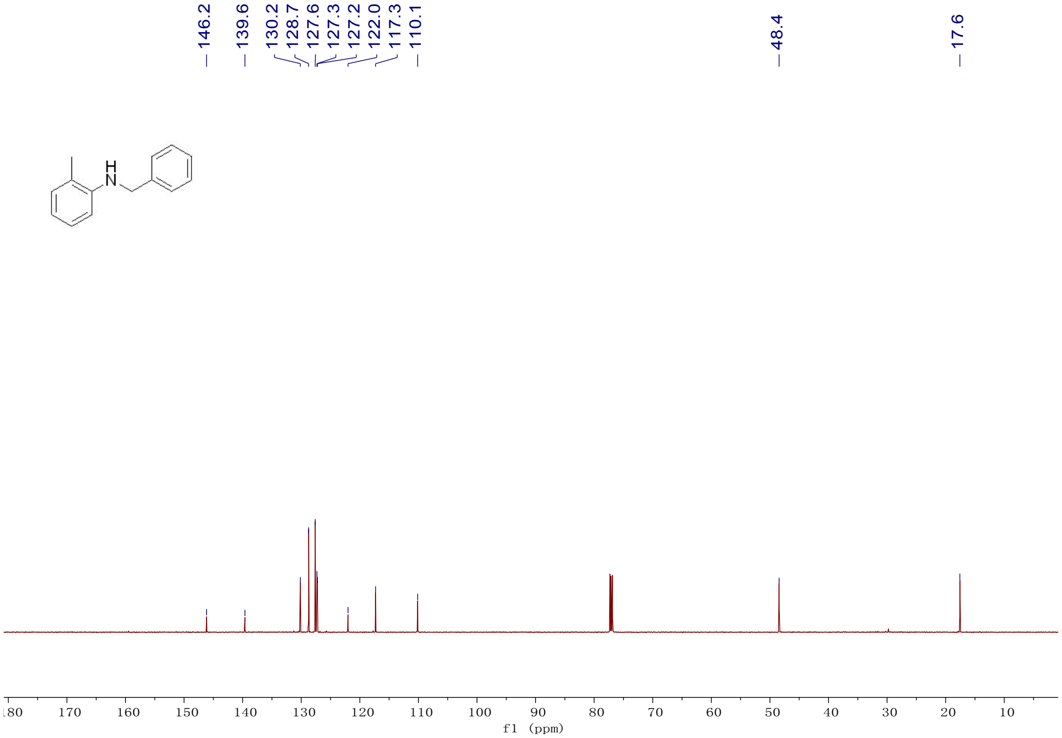


**Figure S36.** $^{13}C\{^{1}H\}$ NMR spectra of **4c** (151 MHz, $CDCl_3$).

***N*-benzyl-4-tert-butylaniline (4d)**

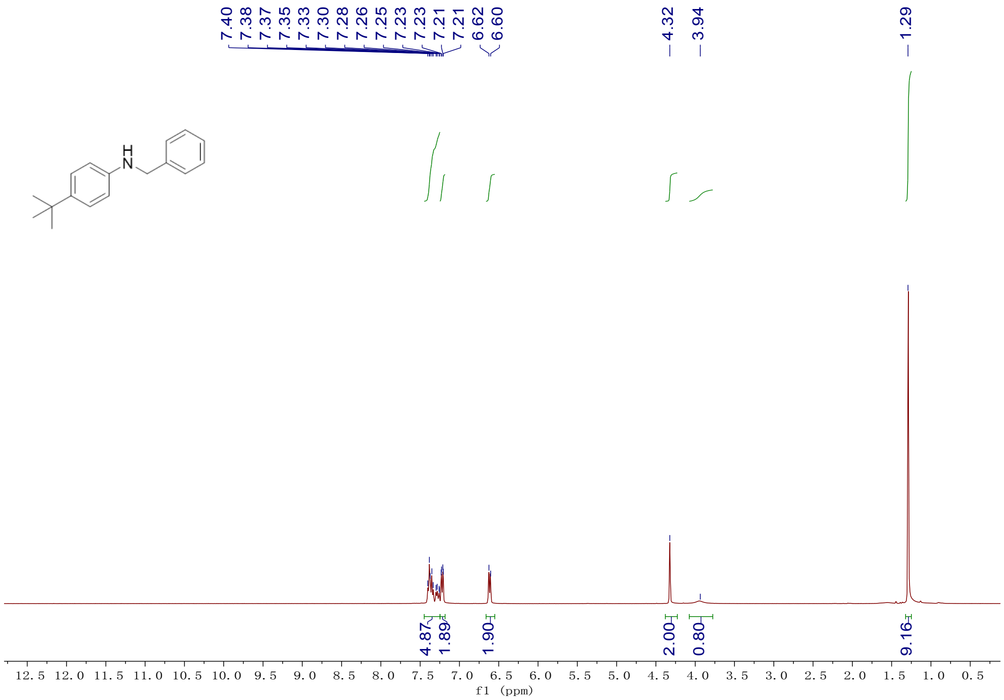


**Figure S37.** $^{1}H$ NMR spectra of **4d** (400 MHz, $CDCl_3$).

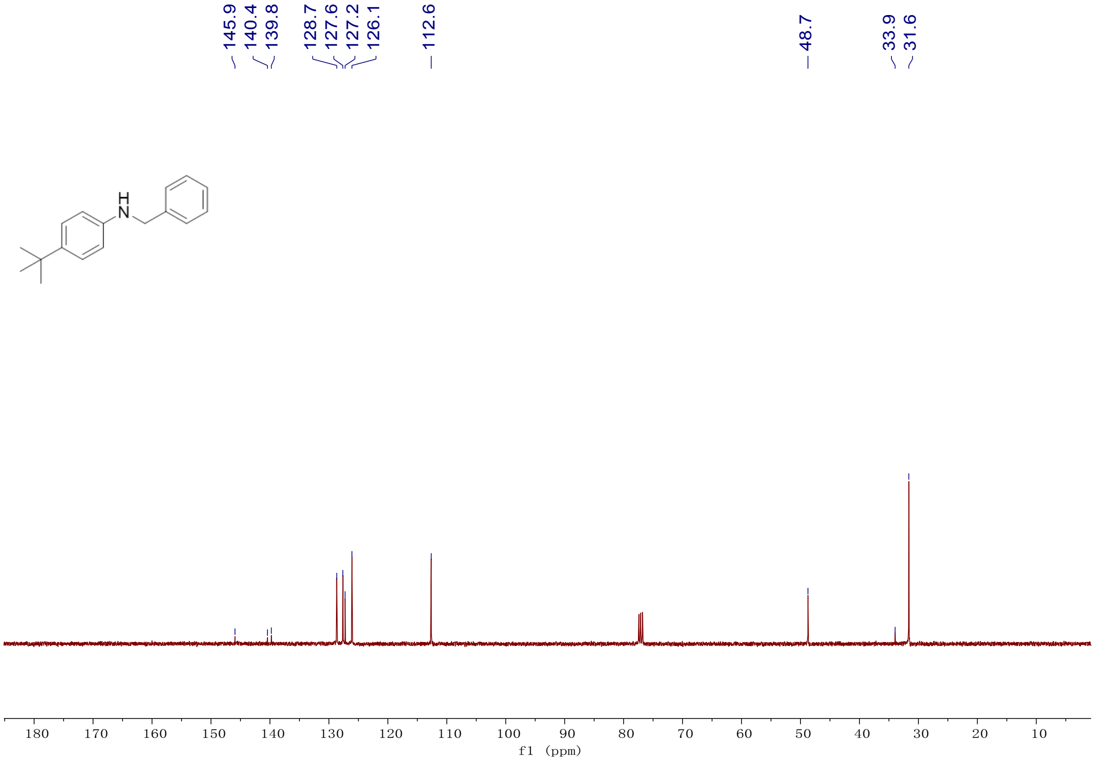


**Figure S38.** $^{13}C\{^{1}H\}$ NMR spectra of **4d** (101 MHz, $CDCl_3$).

***N*-benzyl-4-methoxyaniline (4e)**

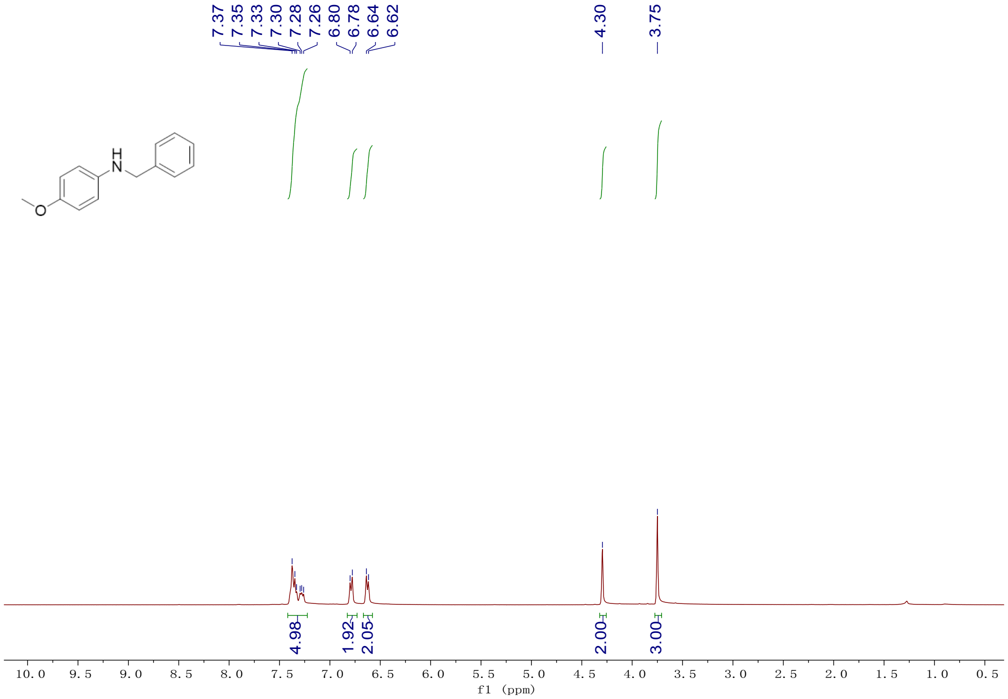


**Figure S39.** $^{1}H$ NMR spectra of **4e** (400 MHz, $CDCl_3$).

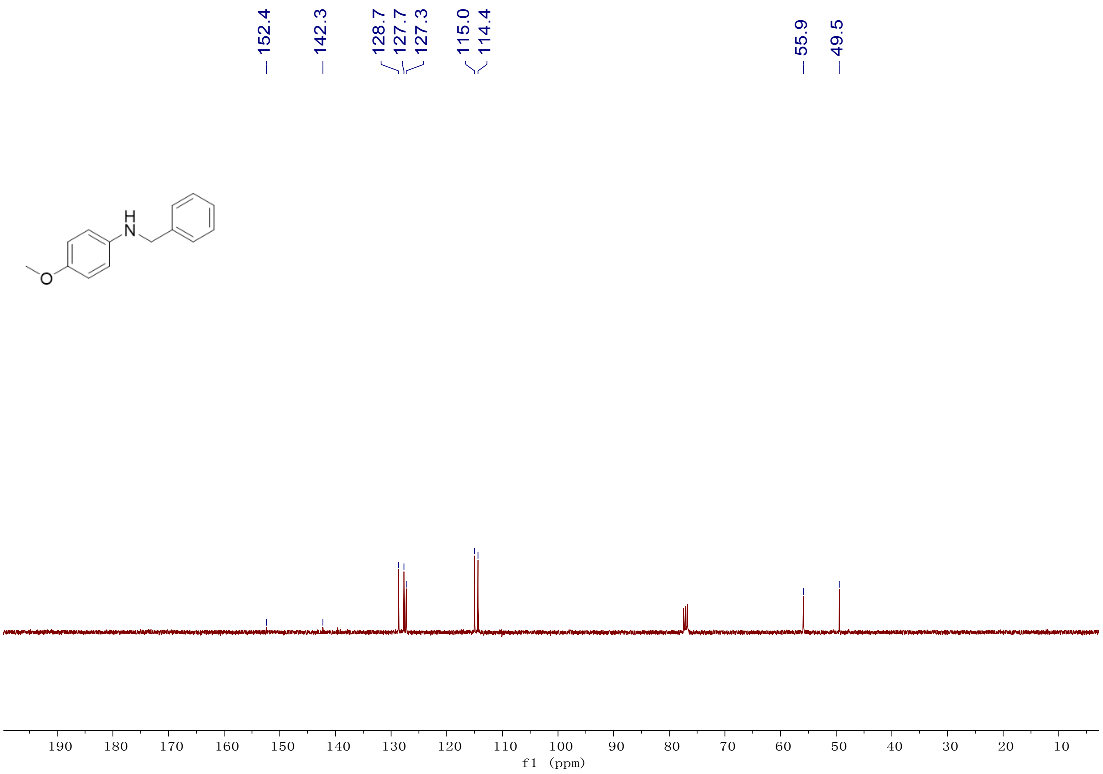


**Figure S40.** $^{13}C\{^1H\}$ NMR spectra of **4e** (101 MHz, $CDCl_3$).

***N*-benzyl-2-methoxyaniline (4f)**

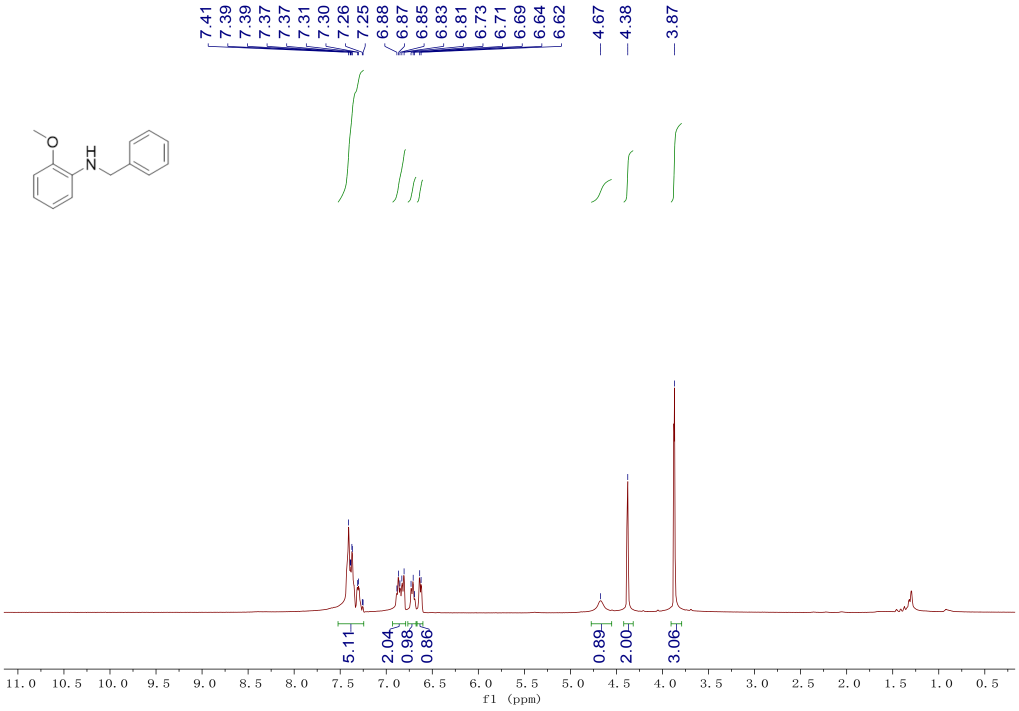


**Figure S41.** $^1H$ NMR spectra of **4f** (400 MHz, $CDCl_3$).

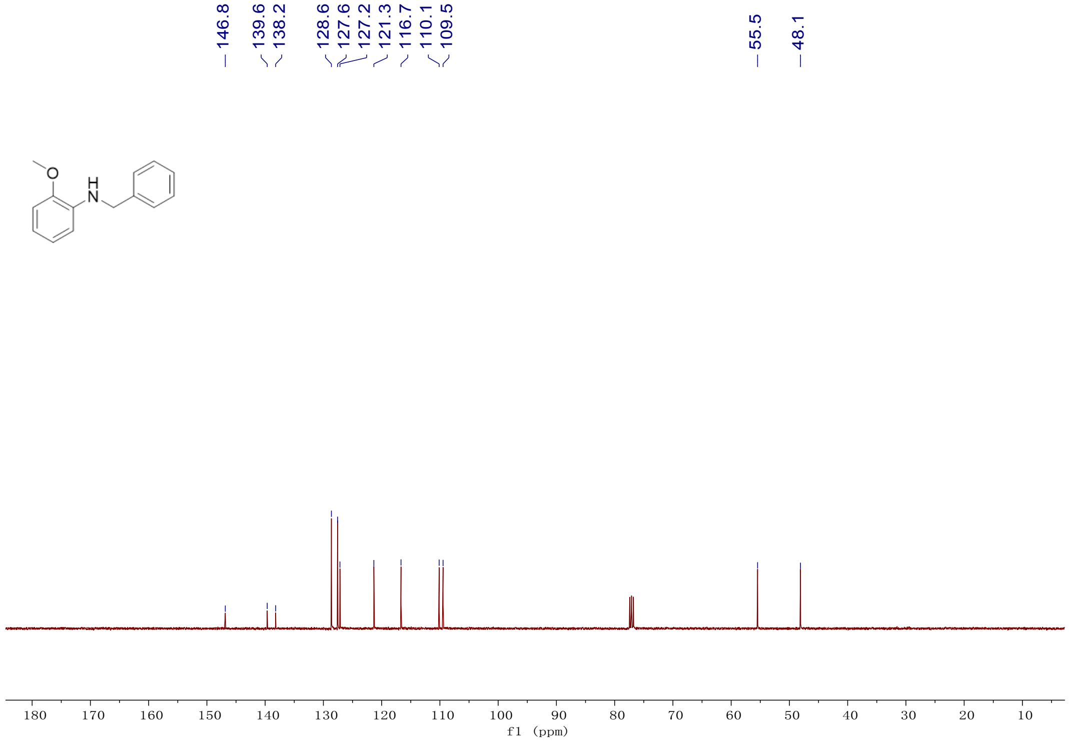


**Figure S42.** $^{13}C\{^{1}H\}$ NMR spectra of **4f** (101 MHz, $CDCl_3$).

## *N*-benzyl-4-fluoroaniline (4g)

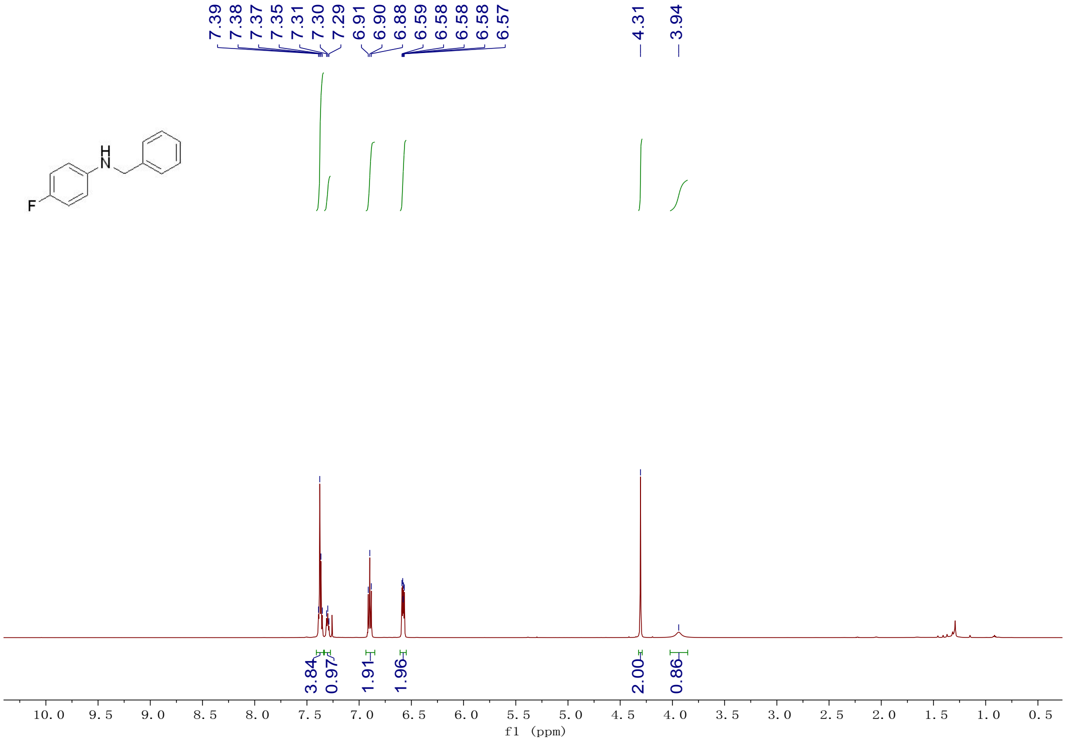


**Figure S43.** $^{1}H$ NMR spectra of **4g** (600 MHz, $CDCl_3$).

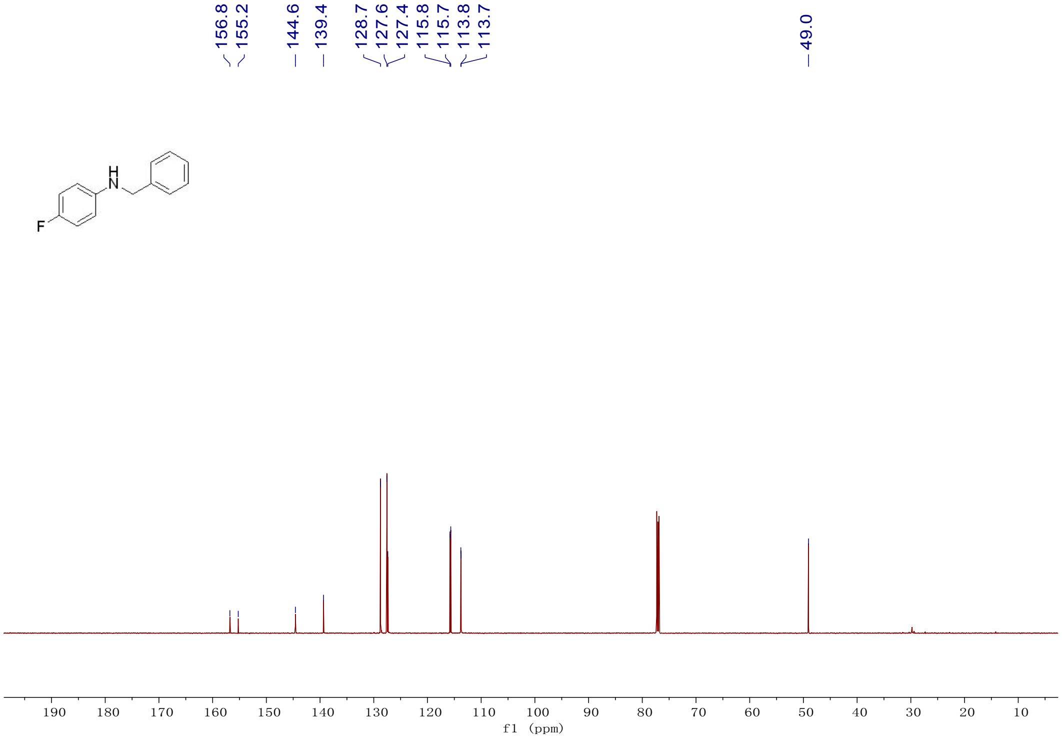


**Figure S44.** $^{13}C\{^{1}H\}$ NMR spectra of **4g** (151 MHz, $CDCl_3$).

***N*-benzyl-4-chloroaniline (4h)**

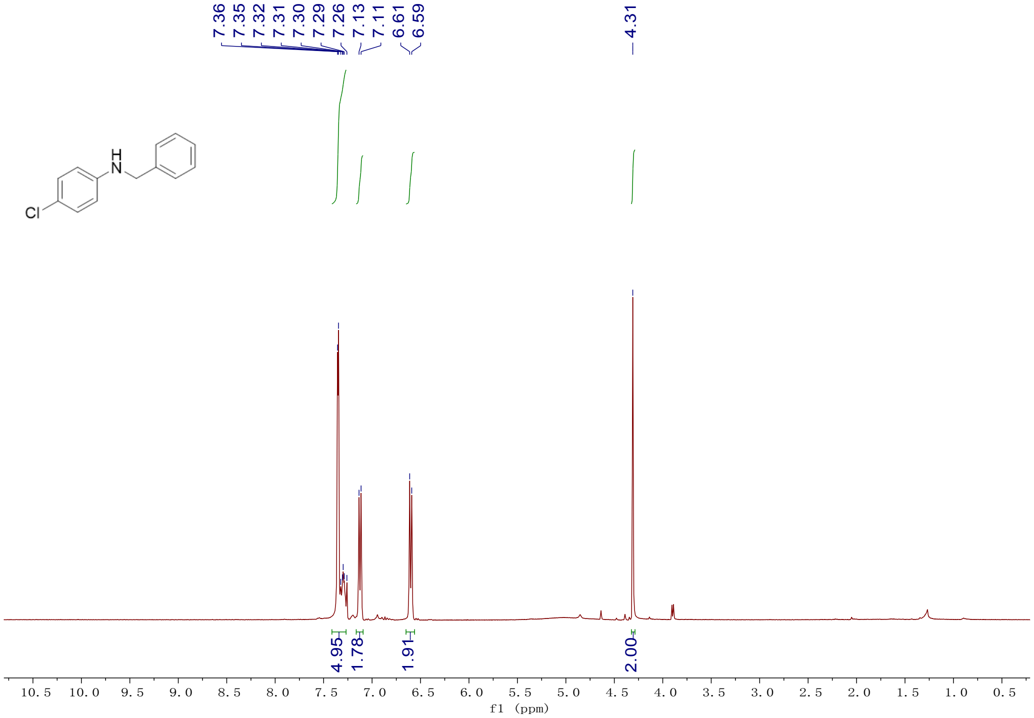


**Figure S45.** $^{1}H$ NMR spectra of **4h** (400 MHz, $CDCl_3$).

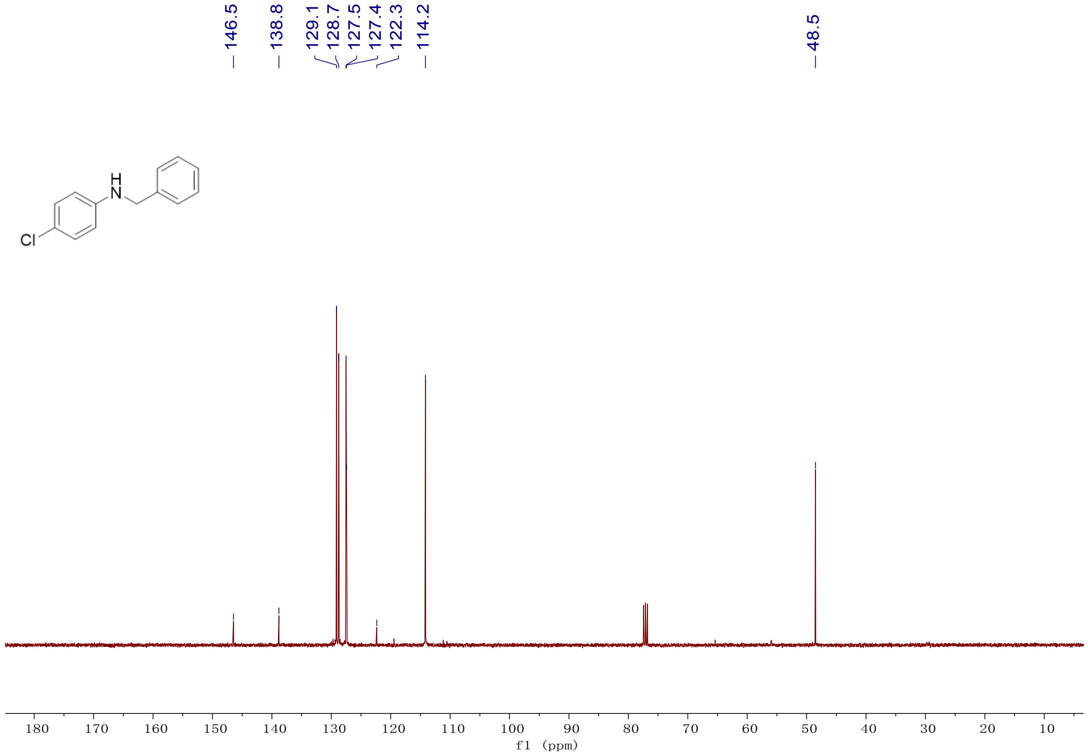


**Figure S46.** $^{13}C\{^{1}H\}$ NMR spectra of **4h** (101 MHz, $CDCl_3$).

## ***N*-benzyl-4-bromoaniline (4i)**

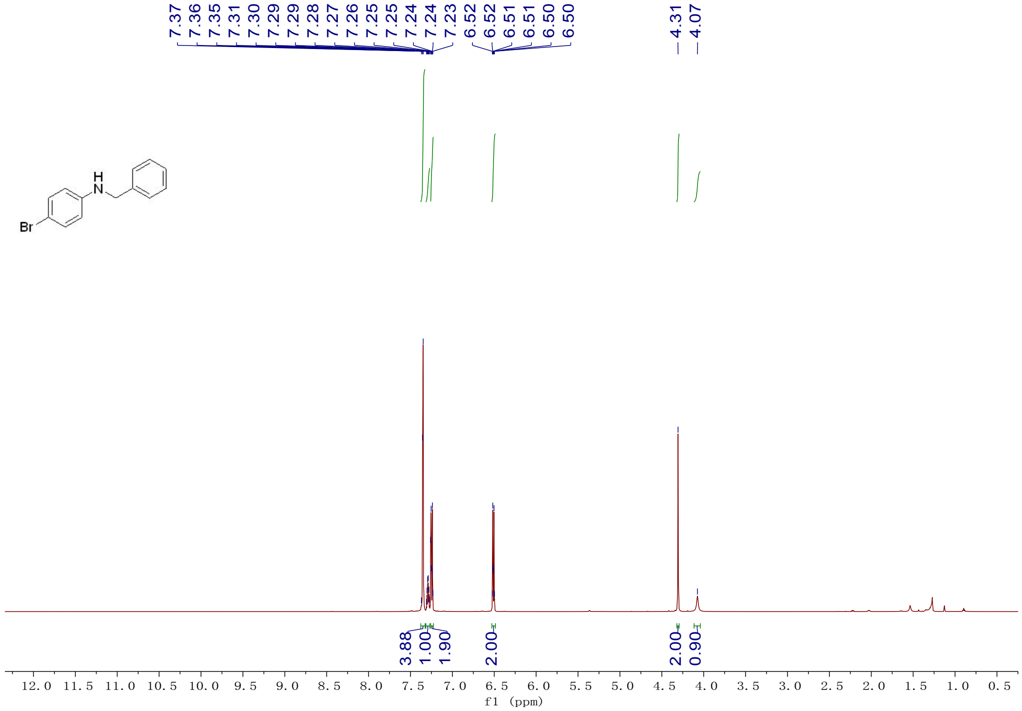

**Figure S47.** ¹H NMR spectra of **4i** (600 MHz, $CDCl_3$).

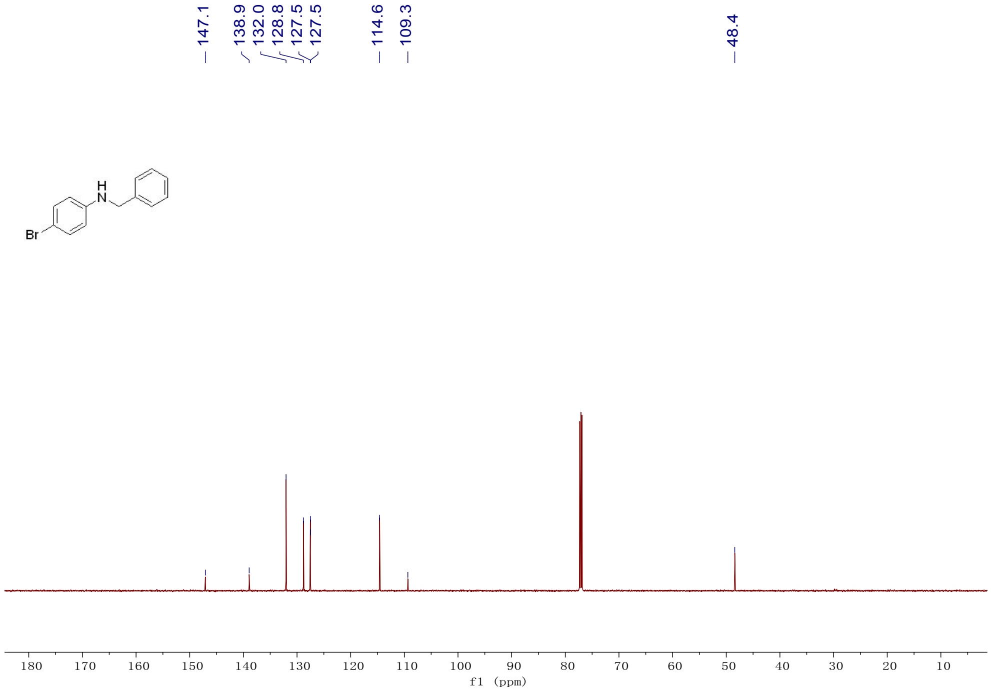


**Figure S48.** $^{13}C\{^1H\}$ NMR spectra of **4i** (151 MHz, $CDCl_3$).

## ***N*, *N′*-dibenzyl-1,2-phenylenediamine (4j)**

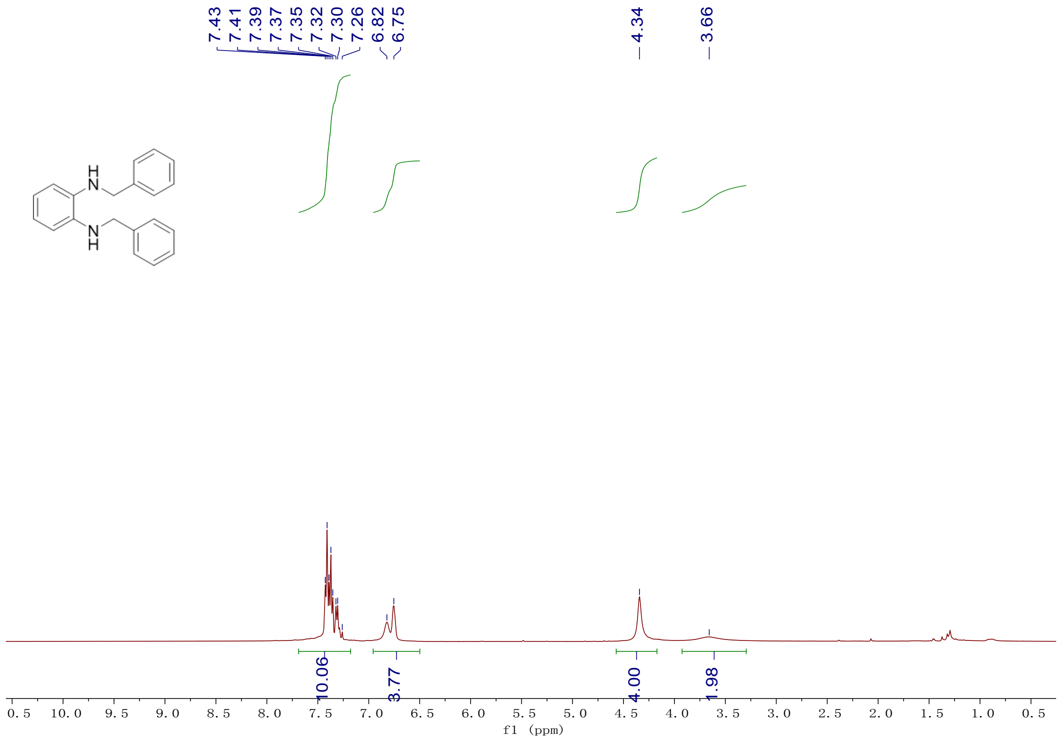

**Figure S49.** $^{1}H$ NMR spectra of **4j** (400 MHz, $CDCl_3$).

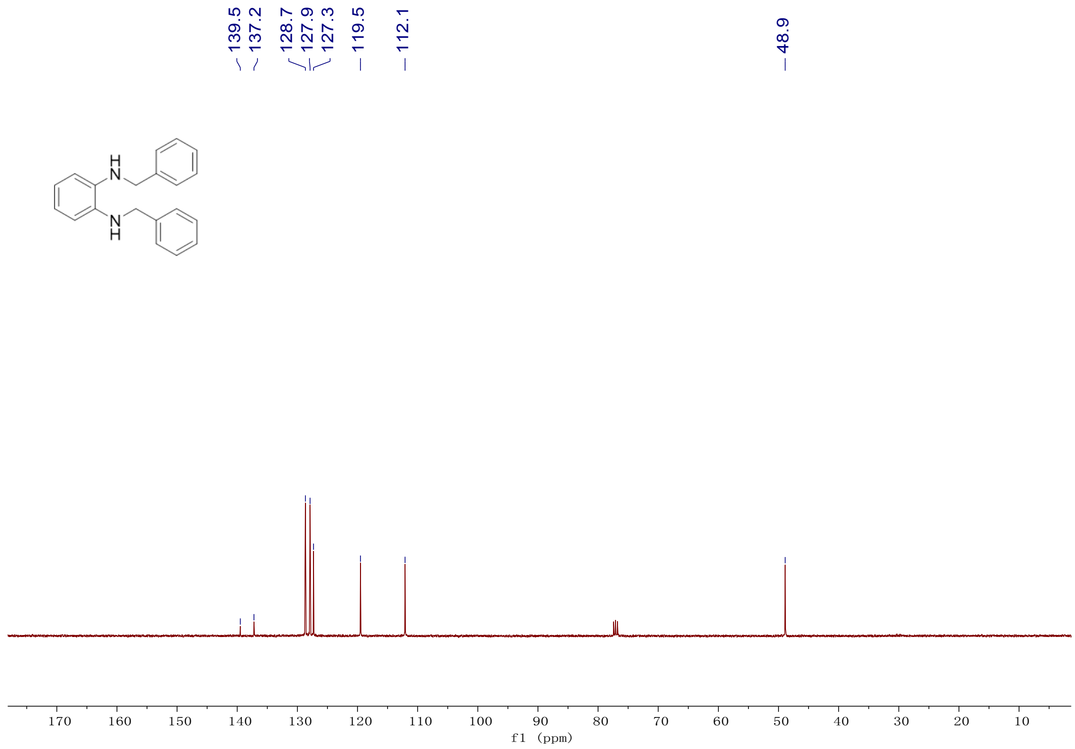


**Figure S50.** $^{13}C\{^{1}H\}$ NMR spectra of **4i** (101 MHz, $CDCl_3$).

# Reference


(1) Homberg, L.; Roller, A.; Hultzsch, K. C. A highly active PN[3] manganese pincer complex performing *N*-alkylation of amines under mild conditions. *Org. Lett.* **2019**, *21* (9), 3142–3147.

(2) Chung, H.; Chung, Y. K. Cobalt-rhodium heterobimetallic nanoparticle-catalyzed *N*-alkylation of amines with alcohols to secondary and tertiary amines. *J. Org. Chem.* **2018**, *83* (15), 8533–8542.

(3) Pan, Y.; Luo, Z.; Xu, X.; Zhao, H.; Han, J.; Xu, L.; Fan, Q.; Xiao, Ru-catalyzed deoxygenative transfer hydrogenation of amides to amines with formic acid/triethylamine. *Adv. Synth. Catal.* **2019**, *361* (16), 3800–3806.

(4) Rösler, S.; Ertl, M.; Irrgang, T.; Kempe, R. Cobalt-catalyzed alkylation of aromatic Amines by alcohols. *Angew. Chem. Int. Ed.* **2015**, *54* (50), 15046–15050.

(5) Xu, Q.; Xie, H.; Zhang, E.-L.; Ma, X.; Chen, J.; Yu, X.-C.; Li, H. Selective catalytic hofmann *N*-alkylation of poor nucleophilic amines and amides with catalytic amounts of alkyl halides. *Green Chem.*, **2016**, *18*, 3940-3944.

(6) Yuan, M.-L.; Xie, J.-H.; Zhu, S.-F.; Zhou, Q.-L. Deoxygenative hydrogenation of amides catalyzed by a well-defined iridium pincer complex. *ACS Catal.* **2016**, *6* (6), 3665–3669.

(7) Li, Q.-Q.; Xiao, Z.-F.; Yao, C.-Z.; Zheng, H.-X.; Kang, Y.-B. Direct alkylation of amines with alcohols catalyzed by base. *Org. Lett.* **2015**, *17* (21), 5328–5331.
(8) Yang, W.; Wei, L.; Yi, F.; Cai, M. Magnetic nanoparticle-supported phosphine gold(I) complex: a highly efficient and recyclable catalyst for the direct reductive amination of aldehydes and ketones. *Catal. Sci. Technol.* **2016**, *6* (12), 4554–4564.
(9) Fan, L.; Jia, J.; Hou, H.; Lefebvre, Q.; Rueping, M. Decarboxylative aminomethylation of aryl- and vinylsulfonates through combined nickel- and photoredox-catalyzed cross-coupling. *Chem. Eur. J.* **2016**, *22* (46), 16437–16440.
(10) Wagner, P.; Bollenbach, M.; Doebelin, C.; Bihel, F.; Bourguignon, J.-J.; Salomé, C.; Schmitt, M. t-BuXPhos: a highly efficient ligand for Buchwald-Hartwig coupling in water. *Green Chem.* **2014**, *16* (9), 4170–4178.
(11) Huang, Z.; Wang, S.; Zhu, X.; Yuan, Q.; Wei, Y.; Zhou, S.; Mu, X. Well-defined amidate-functionalized *N*-heterocyclic carbene (NHC) supported rare earth metal complexes as catalysts for efficient hydroboration of unactivated imines and nitriles. *Inorg. Chem.* **2018**, *57* (24), 15069–15078.
(12) Zhou, W.; Fan, M.; Yin, J.; Jiang, Y.; Ma, D. CuI/oxalic diamides catalyzed coupling reaction of (hetero)aryl chlorides and amines. *J. Am. Chem. Soc.* **2015**, *137* (37), 11942–11945.
(13) Qian, C.; Tang, W. A versatile synthesis of vinyl-substituted heterocycles via regio- and enantioselective Pd-catalyzed tandem allylic substitution. *Org. Lett.* **2020**, 22 (11), 4483–4488.
